# Exploring the Use of “Honorary Transition Metals” To Push the Boundaries of Planar Hypercoordinate Alkaline-Earth Metals

**DOI:** 10.1021/jacs.4c03977

**Published:** 2024-06-06

**Authors:** Xin-bo Liu, William Tiznado, Li-Juan Cui, Jorge Barroso, Luis Leyva-Parra, Lin-hong Miao, Hui-yu Zhang, Sudip Pan, Gabriel Merino, Zhong-hua Cui

**Affiliations:** † Institute of Atomic and Molecular Physics, Jilin University, Changchun 130023, China; ‡ Centro de Química Teórica & Computacional (CQT&C), Facultad de Ciencias Exactas, Departamento de Ciencias Químicas, Universidad Andrés Bello, Avenida República 275, 8370146 Santiago de Chile, Chile; § Department of Chemistry, Clemson University, Clemson, South Carolina 29634, United States; ∥ Departamento de Física Aplicada, Centro de Investigación y de Estudios Avanzados Unidad Mérida, Km 6 Antigua Carretera a Progreso. Apdo. Postal 73, Cordemex, 97310 Mérida, México; ⊥ Key Laboratory of Physics and Technology for Advanced Batteries (Ministry of Education), Jilin University, Changchun 130023, China

## Abstract

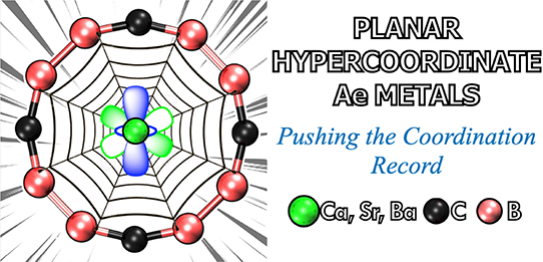

The quest for planar hypercoordinate atoms (phA) beyond six has predominantly focused on transition metals, with dodecacoordination being the highest reported thus far. Extending this bonding scenario to main-group elements, which typically lack d orbitals despite their larger atomic radius, has posed significant challenges. Intrigued by the potentiality of covalent bonding formation using the d orbitals of the heavier alkaline-earth metals (Ae = Ca, Sr, Ba), the so-called “honorary transition metals”, we aim to push the boundaries of planar hypercoordination. By including rings formed by 12–15 atoms of boron–carbon and Ae centers, we propose a design scheme of 180 candidates with a phA. Further systematic screening, structural examination, and stability assessments identified 10 potential clusters with a planar hypercoordinate alkaline-earth metal (phAe) as the lowest-energy form. These unconventional structures embody planar dodeca-, trideca-, tetradeca-, and pentadecacoordinate atoms. Chemical bonding analyses reveal the important role of Ae d orbitals in facilitating covalent interactions between the central Ae atom and the surrounding boron–carbon rings, thereby establishing a new record for coordination numbers in the two-dimensional realm.

## Introduction

For decades, chemists have been captivated by planar hypercoordinate atoms (phA).^1−5^ These intriguing motifs question some of the well-established bonding rules. For instance, planar tetracoordinate carbons (ptC)^6^ challenge the longstanding tetrahedral carbon model, a paradigm in organic chemistry. Although ptCs emerged on paper,^7,8^ some were later synthesized in bottleable quantities^9−16^ or were detected in the gas phase,^17−23^ suggesting that specific geometrical and electronic criteria are needed for their materialization. As a result, some design principles were developed such as the 18-valence electron counting rule.^24−32^ This rule plays a fundamental role in predicting these fascinating molecules and helps to expand the concept of phAs beyond carbon to include all main-group elements^33,34^ in the second period, except Ne, and many other elements from the subsequent periods.^35−40^

As expected, maintaining planarity becomes gradually challenging as the coordination number increases. This is evidenced by the relative abundance of planar tetracoordinate atoms in comparison to the relative scarcity of examples of planar higher-coordinate atoms. Planar hexacoordinate atom motifs are the current record for carbon,^41^ while transition-metal-centered boron structures show higher coordination numbers.^42−45^ Some thermodynamically favorable examples with a phA are Co©B_8_^–^,^46^ Fe©B_9_^–^,^47^ Ru©B_9_^–^,^46^ Rh©B_9_,^48^ Ir©B_9_,^48^ Nb©B_10_^–^,^49^ Ta©B_10_^–^,^49^ Y©B_8_C_4_,^50^ and La©B_8_C_4_^–^.^51^ The relatively high stability of these transition-metal-centered structures is attributed to σ- and π-double aromaticity, which arises primarily from the partially filled d orbitals of the transition metals and the delocalized multicenter σ- and π-bonds of the boron rings. The participation of d orbitals is crucial, as boron wheels with a main group element as a centered atom are not the most energetically favorable isomers,^52−55^ despite following similar electron counting rules. This is evident in the case of AlB_7_^–^ and AlB_8_^–^ clusters, which prefer umbrella-type structures over molecular wheels.^56^

Recent studies have emphasized the significance of d orbitals in covalent interactions in heavier alkaline-earth elements (Ae = Ca, Sr, and Ba).^57,58^ Their large atomic radii and transition metal-like behavior play a key role in our efforts to achieve energetically viable planar hypercoordinate atoms with higher coordination numbers. By using binary boron–carbon rings, we designed 180 combinations with the formula AeB_*x*_C_*y*_^*q*^ (12 ≤ *x* + *y* ≤ 15; *q* = 0, ±1, *n* > 0). Ten species with planar hypercoordinate alkaline-earth atoms, namely, Ca©B_8_C_4_^–^, Ca©B_7_C_5_^–^, Ca©B_6_C_6_^–^, Ca©B_6_C_6_, Ca©C_13_,^59^ Sr©B_8_C_5_^–^, Sr©BC_13_^+^, Ba©B_4_C_10_^–^, Ba©B_3_C_12_^+^, and Ba©B_2_C_13_^+^, were identified as the global minima on their corresponding potential energy surfaces (PESs). The involvement of Ae d orbitals in delocalized σ- and π-bonding leads to strong covalent interactions between the central Ae atom and peripheral ring. These putative global minima represent a milestone in the field of phA by setting a new record for the highest coordination number in 2D space, reaching planar dodeca-, trideca-, tetradeca-, and pentadecacoordinate atoms.

## Results and Discussion

### High-Throughput Screening of Planar Hypercoordinate Alkaline-Earth Metals (phAe)

To successfully design viable planar hypercoordinate alkaline-earth metal species, two conditions must be met: (i) a precise balance between the geometric and electronic criteria and (ii) the arrangements must be energetically favorable and not prone to isomerize. We have worked on binary boron–carbon rings to overcome the unstable nature of pure boron rings. The present approach involves 486 combinations of the formula AeB_*x*_C_*y*_^*q*^, where the sum of *x* and *y* ranges from 12 to 15 and includes cationic, neutral, and anionic charge states (*q* = 0, ±1). A key element in our strategy was to guarantee the best possible geometrical coupling between the central atom (Ca, Sr, or Ba) and the peripheral ring. In other words, we look to ensure a perfect fit between the central atom radius and the outer radial orbital distribution without compromising the covalent bond. For example, because of the smaller sizes of carbon than boron, the smaller cavities are formed by pure carbon rings than the corresponding pure boron and binary boron–carbon rings. Our calculations indicate that a pure carbon ring such as C_16_ is too large to accommodate the Ba atom. Therefore, a selective addition of boron atoms in the carbon ring will increase the radius of the peripheral ring, and eventually, it might lead to stable phAs with a maximum coordination number of 15.

**Scheme 1:**
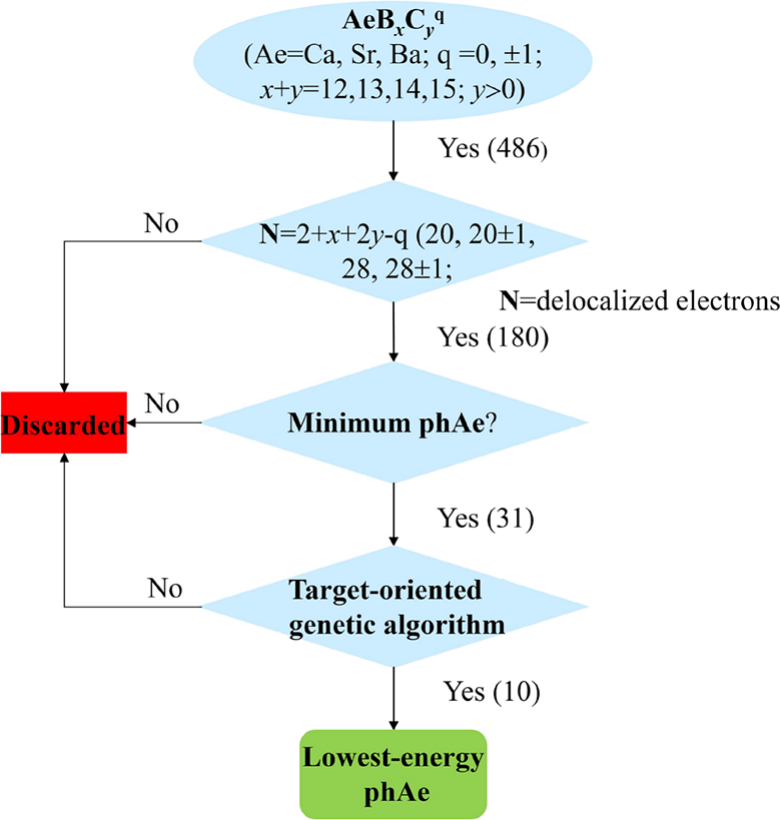
Workflow Chart for Exploring the Lowest Energy phAe (Ae = Ca, Sr, or Ba) Species

In addition, aromaticity is known to play a key role in stabilizing such planar hypercoordinate species. Let us explore the possibility of inducing double (σ- and π-) aromaticity in these systems.^60^ Note that for a coordination number greater than 10, the number of delocalized σ- and π-orbitals is equal. According to Hückel’s rule and considering the number of atoms constituting the rings, at least 10 σ- and 10 π-orbitals are required to be filled. The other suitable setting would involve 56 electrons in 14 σ- and 14 π-orbitals. Using this electron counting rule, we derived the expression 2 + *x* + 2*y* – *q*, where *x*, *y*, and *q* represent the electrons and charge of clusters with the chemical formula AeB_*x*_C_*y*_^*q*^, i.e., Ae contributes two valence electrons, B contributes three, two of which are involved in peripheral bonding (thus contributing one electron each to delocalized bonding), and carbon contributes four, two of which are engaged in peripheral bonding and two are available for delocalized bonding. This count limits our candidate selection to 180 (as indicated in Scheme 1 and Table S1), with the −CBC– and −BBC– structures forming the peripheral rings.

The PESs of these 180 combinations were explored using a target-oriented genetic algorithm.^61^ Geometric optimizations were carried out using M06-2X^62^ and PBE0^63^-D3^64^ functionals in conjunction with the def2-TZVP^65^ basis set. The algorithm compares the lower energy isomers with the designed “target” structure, continuing until either convergence or a predetermined number of iterations is reached. For those combinations that find a global minimum with a phAe, an additional search was conducted using CALYPSO^66^ at the M06-2X/def2-TZVP level (Table S2).

Table S1 summarizes the results for the 180 phAe species. In the cases where the sum of *x* and *y* in B_*y*_C_*x*_ rings is greater than 13, calcium clusters have a noticeable structural distortion, suggesting that the cavity size is too large to accommodate a Ca atom adequately. In contrast, imaginary frequencies correspond to out-of-plane modes for Sr and Ba clusters with *x* + *y* ≤ 13, indicating that the cavity is too small to fit these atoms. These findings underscore the importance of a precise geometric match between the central atom and the peripheral rings, as stated in condition (i).

### Structures and Stabilities of phAe

**Figure 1:**
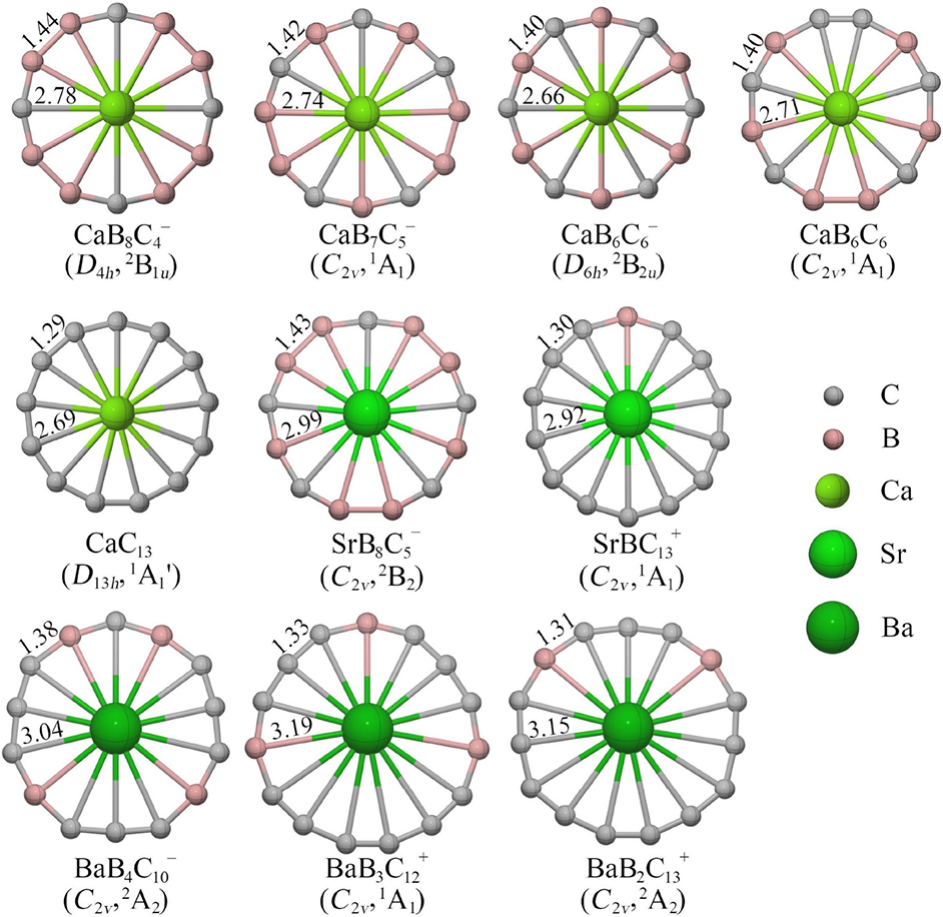
M06-2X/def2-TZVP structures of the lowest-energy phAe of CaB_8_C_4_^–^, CaB_7_C_5_^–^, CaB_6_C_6_^–^, CaB_6_C_6_, CaC_13_^–^, SrB_8_C_5_^–^, SrBC_13_^+^, BaB_4_C_10_^–^, BaB_3_C_12_^+^, and BaB_2_C_13_^+^. The average bond distances of peripheral ligand rings and center ligands are given in Å.

The lowest-energy phAe structures obtained through our approach and optimized at the M06-2X/def2-TZVP level were further validated using single-point computations using the CCSD(T)^67^ methodology. Our analysis confirmed these phAe isomers as the most energetically favorable species for their respective stoichiometries (Figure 1). Figures S1–S9 show various low-lying isomers of these systems with a phAe, excluding CaC_13_, which has already been reported.^59^ At the CCSD(T)/def2-TZVP//M06-2X/def2-TZVP level, several low-lying energy isomers are phAe clusters. For CaB_8_C_4_^–^, BaB_4_C_10_^–^, BaB_3_C_12_^+^, and BaB_2_C_13_^+^, the second lowest energy forms, which are close in energy to the corresponding global minimum (0.1 to 1.3 kcal/mol), is a phAe. In contrast, systems like CaB_7_C_5_^–^, CaB_6_C_6_^–^, CaB_6_C_6_, and SrB_8_C_5_^–^ show larger energy differences (ranging from 3.5 to 7.3 kcal/mol) between the phAe structures (the global minima) and the second-lowest energy forms. Interestingly, the phAe isomers of CaC_13_ and SrBC_13_^+^ exhibit a significant energy gap with their nearest energy isomers. For example, the phAe configuration of SrBC_13_^+^ is 41.8 kcal/mol lower in energy than its closest isomer. We also explored PESs with higher multiplicities, but all the local minima in these states are higher in energy than the singlet state structures. In addition, the *T*_1_ diagnostic^68^ values, as detailed in Figures S1–S9, support the suitability of monodeterminantal methods for analyzing these phAe species.

Born–Oppenheimer molecular dynamics (BO-MD) simulations were conducted at 400 K to evaluate the kinetic stability of the putative global minima with a phAe (Figure S10). Throughout the simulations, we noted that the structural integrity and planarity of these systems were maintained, showing no signs of isomerization or other structural changes. This was corroborated by minimal root-mean-square deviation values (Figure S10). During these BO-MD simulations, a connection between bond stretching and the lowest vibrational modes was noted, especially the out-of-plane mode of the central Ae atom. The energy curves for the out-of-plane displacement of the central Ae atom highlight a strong preference for planar geometries over three-dimensional structures (Figure S11). Therefore, the thermodynamic and kinetic stabilities of the structures with phAe indicate their viability for experimental detection. To facilitate the experimental validation, we provide simulated photoelectron spectra for anionic systems and infrared spectra for neutral and cationic systems in Figure S12.

To quantify the stability of the phAe clusters in terms of Ae dissociation, we have computed the energy of three dissociation pathways where the B_*x*_C_*y*_ rings are retained after removing the central Ae atom (see Table S3). The proposed dissociation channels are

Pathway I: AeB_*x*_C_*y*_^*q*^ → Ae + B_*x*_C_*y*_^*q*^;
Pathway II: AeB_*x*_C_*y*_^*q*^ → Ae^+^ + B_*x*_C_*y*_^*q*–1^;
Pathway III: AeB_*x*_C_*y*_^*q*^ → Ae^2+^ + B_*x*_C_*y*_*^q^*^–2^;

Pathway I, which leads to the detachment of the neutral Ae atom, exhibits the lowest bond dissociation energy (Δ*E*_diss_) values in the cases of neutral and anionic phAe species. The large Δ*E*_diss_ values ranging between 111.5 kcal/mol for Ca©B_8_C_4_^–^ and 193.6 kcal/mol for Ca©B_7_C_5_^–^ indicate considerable stability of such phAe clusters toward Ae detachment. For cationic phAe species, Pathway II represents the most readily dissociation, which has Δ*E*_diss_ values between 79.5 (Ba©B_2_C_13_^+^) and 121.8 (Ba©B_3_C_12_^+^) kcal/mol. These dissociation values suggest strong bonding interactions between the phAe centers and the peripheral B_*x*_C_*y*_ rings. Recent theoretical explorations of alkali-metal-centered carbon rings, such as Na©C_14_^+^ and Cs©C_18_^+^, revealed planar hypercoordinate alkali metals primarily stabilized by weak interactions, as evidenced by their small cationic alkali-metal dissociation energies (22.7 and 15.2 kcal/mol, respectively).^69^ Undoubtedly, from the stability aspect of large planar hypercoordinate systems, alkaline-earth atoms have an upper hand over alkali-metals as the latter only can interact through electrostatic interaction, whereas the former ones can employ both electrostatic and covalent interactions with peripheral rings incorporating larger stability (*vide infra*).^70^

Population analyses, using natural population analysis (NPA),^71^ CM5,^72^ Hirshfeld,^73^ Bader,^74^ and Voronoi^75^ charges of the global minima containing a phAe indicate a positive charge on the phAe atom, as shown in Table S4. Note that the partial charges computed with different approximations give significantly different values. Specifically focusing on the charge on Ae (*Q*_Ae_), NPA and Bader charges generally show a very large positive charge (1.53–1.91 *e*), while the Hirshfeld and Voronoi charges yield smaller positive charges (0.45–0.89 *e*). The CM5 method, on the other hand, gives values in between (1.07–1.26 *e*). Why? Natural bond order approach considers only *n*(s) orbital of Ae atoms as valence orbitals, and *n*(p) and (*n –* 1)d orbitals as Rydberg orbitals. In a subsequent step, it gives less weightage to the Rydberg orbitals, prioritizing only the valence orbitals. Such preselection sometimes could give biased results, particularly for the cases where those considered Rydberg orbitals take part dominantly in the bonding.^76,77^ For the present cases, (*n* – 1)d and *n*(p) orbitals of the Ae atom are even more important in the bonding than the *n*(s) orbital. NPA overestimates the positive charge on it. Similarly, the Bader approach, which uses a zero-flux surface to partition atomic regions and then integrates over those regions, is known to give a strong ionic character of a bond.^75^ The Hirshfeld alternative exhibits variability in reproducing molecular dipole moments and electrostatic potentials, making the CM5 charges a more reasonable choice as it is a parametrized version of the Hirshfeld one to reproduce the molecular dipole moments. In a benchmarking study of atomic partial charges, Cho et al.^78^ noted that “no single charge distribution tells the whole story”. Nevertheless, the charge distribution indicates significant net electron donation from Ae atoms to the B_*x*_C_*y*_ ring, thus promoting a strong electrostatic interaction between Ae and B_*x*_C_*y*_ motifs and contributing to the elevated dissociation energies in Pathways I and II.

As shown in Figure 1, the average bond lengths within the peripheral boron–carbon rings range from 1.55 to 1.59 Å for B–B bonds, 1.38 to 1.41 Å for B–C bonds, and 1.23 to 1.29 Å for C–C bonds. These bond lengths are significantly shorter than the single bond lengths of B–B (1.70 Å), B–C (1.60 Å), and C–C (1.50 Å) based on covalent radii reported by Pyykkö and Atsumi.^79^ Moreover, the Mayer bond order (MBO)^80^ values, approximately 1.5 for B–B, B–C, and C–C bonds, hint at a multiple-bond character (see Table S5). The Ae–B bond distances are approximately 2.66–2.80, 2.92–3.05, and 3.00–3.26 Å, for Ca, Sr, and Ba, respectively, whereas the Ae–C bond distances range from 2.69 to 2.77 Å for Ca, 2.79 to 3.09 Å for Sr, and 2.91 to 3.37 Å for Ba. These Ae–B and Ae–C bond distances are somewhat longer than the sum of the covalent radii of B (0.85 Å), C (0.75 Å), and Ae atoms (1.71 Å for Ca, 1.85 Å for Sr, and 1.96 Å for Ba), which is expected because of the delocalized nature of the Ae–B/C bonding. The MBO values for each Ae–B/C bond range within 0.11–0.15 for dodeca-, 0.08–0.16 for trideca-, 0.05–0.13 for tetradeca-, and 0.03–0.10 for pentadecacoordinate systems.^81^ Given the highly polar Ae–B/C interaction and delocalized bonding, such small values are justified, which gradually reduces to some extent with the increase in the ring size. One exception is BaB_4_C_10_^–^ in which a very negligible MBO value (0.01) is found for two Ae–C^max^ bonds. However, this does not mean that they are not connected, as in subsequent bonding analyses, we found that they are indeed connected via highly delocalized bonds.

### Electron Delocalization and Aromaticity in phAe

**Figure 2:**
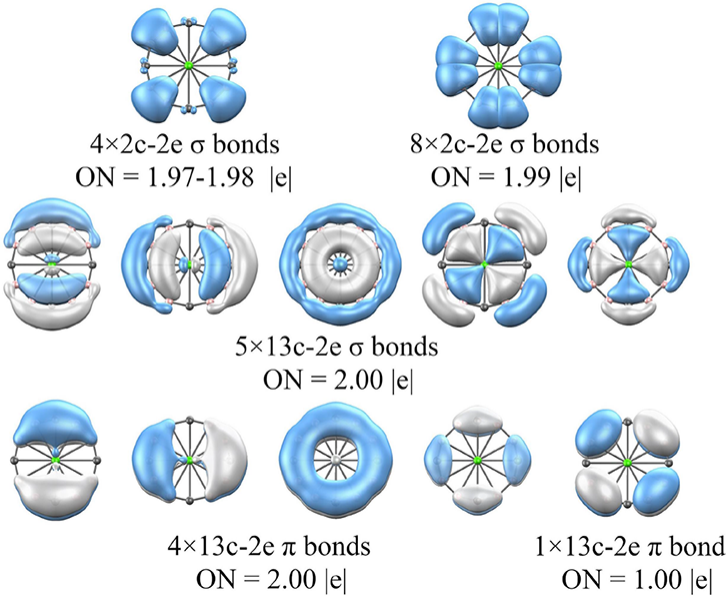
AdNDP analysis of Ca©B_8_C_4_^–^ at the M06-2X/def2-TZVP level. ON stands for occupation number.

In order to gain a better understanding of the bonding and remarkable stability of these phAe clusters, the Adaptive Natural Density Partitioning (AdNDP) analysis^82^ is performed. Due to their similar electronic structures, five representative cases are selected, namely, Ca©B_8_C_4_^–^, Sr©B_8_C_5_^–^, Sr©BC_13_^+^, Ba©B_4_C_10_^–^, and Ba©B_3_C_12_^+^. Figure 2 shows the AdNDP results for Ca©B_8_C_4_^–^, while the other cases are presented in Figures S13–S16. Each species exhibits two sets of bonds. One consists of *n* (where *n* = *x* + *y*) two-center two-electron σ-bonds (2c-2e) that correspond to the localized B–B and B–C covalent bonds (top row of Figure 2). The remaining sets consist of (*n* + 1)c-2e delocalized σ- and π-bonds, representing the delocalized bonding around the boron–carbon ring and the interaction between the Ae atom and the periphery. Notably, this bonding scheme recovers multiple bonding along the B–C ring and the delocalized electrons of (9π + 10σ), (10π + 11σ), (14π + 14σ), (13π + 14σ), and (14π + 14σ) for the Ca©B_8_C_4_^–^, Sr©B_8_C_5_^–^, Sr©BC_13_^+^, Ba©B_4_C_10_^–^, and Ba©B_3_C_12_^+^ phAe clusters, respectively. A key finding is the role of the (*n* – 1)d atomic orbitals of Ca, Sr, and Ba in the delocalized σ- and π-orbitals. Therefore, this bonding analysis strongly validates our design strategy, which takes advantage of a double-aromaticity electron counting system to facilitate delocalized bonding.

**Figure 3:**
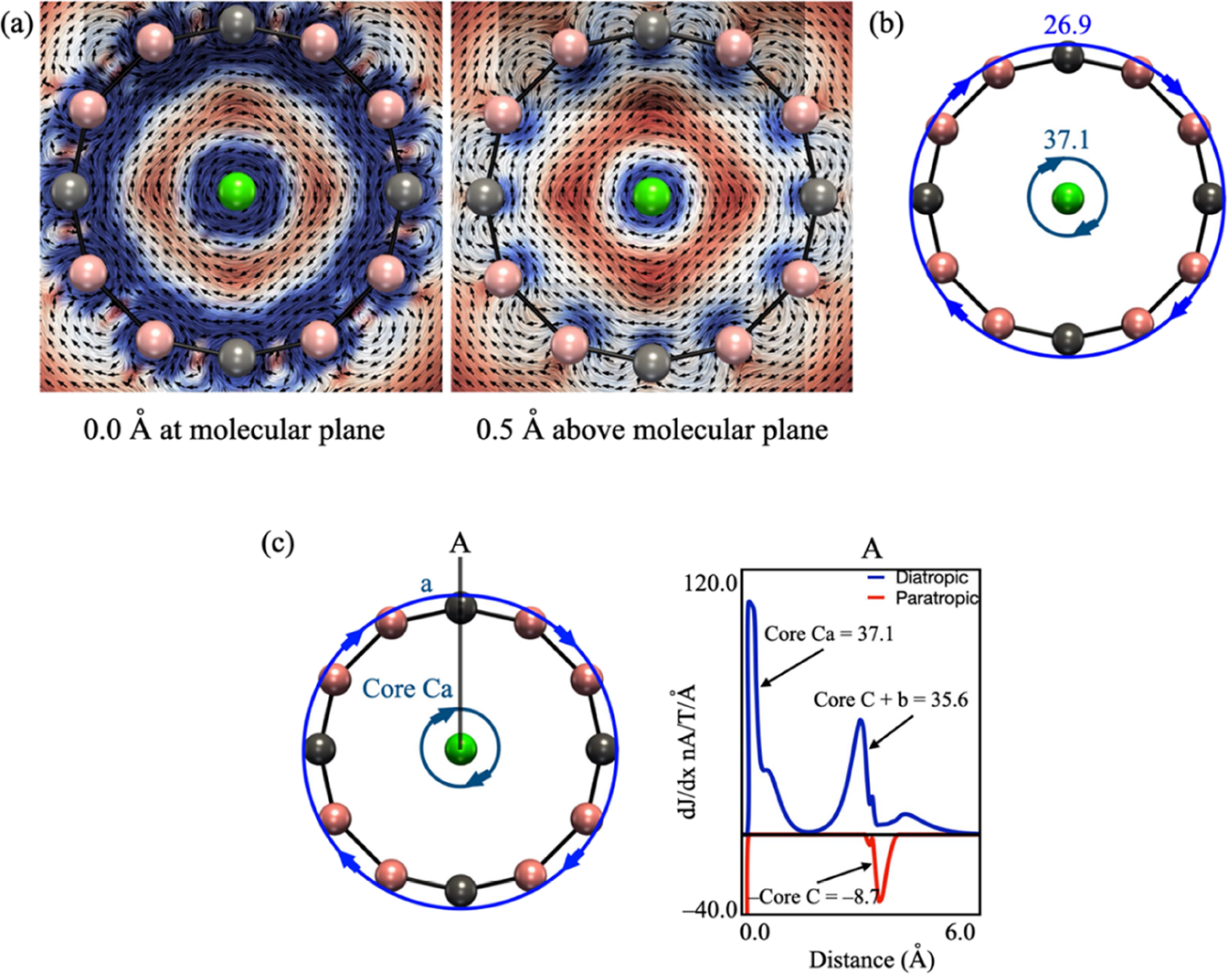
Analysis of magnetically induced current density for Ca©B_8_C_4_^–^. (a) Plot of vectors at 0.0 and 0.5 Å on the molecular plane. (b) Schematic representation of the ring currents and their strengths in nA/T. (c) Integration plane used in the analysis and the integration profile resulted from this plane. The numbers in each band (integrated values of the flux in nA/T) identify the different contributions.

So far, our results strongly suggest high electronic delocalization in these structures. It is worth mentioning that there is one extra electron for the doublet systems than Hückel’s (4*n* + 2) rule of aromaticity. For these systems, the presence of single-electron delocalized orbitals may influence the total aromaticity. To explore this characteristic further, we have carried out a ring current analysis through the gauge-including magnetically induced current (GIMIC) program (see Figure 3 for Ca©B_8_C_4_^–^ and Figures S17–S24 for others).^83^ In aromatic molecules, an external magnetic field perpendicular to the molecular plane induces a diatropic (clockwise) ring current. The vector plots at 0.0 and 0.5 Å above the molecular plane show that both the inner and outer portions of the peripheral ring display a diatropic and unidirectional current, indicative of aromaticity. This double aromaticity (σ + π) differs from the typical π-aromaticity of benzene. Note that in our present cases, five systems (Ca©B_8_C_4_^–^, Ca©B_6_C_6_^–^, Ba©B_2_C_13_^–^, Sr©B_8_C_5_^–^, and Ba©B_4_C_10_^–^) are doublets. The first three still display diatropic ring currents both at the molecular plane and 0.5 Å above it (see Figures 3, S17, and S23). In contrast, Ba©B_4_C_10_^–^ shows a paratropic ring current at the plane 0.5 Å above the molecular plane, presumably due to its odd-electron located in a π-orbital (Figures S15 and S22), while Sr©B_8_C_5_^–^ has the odd-electron in a σ-orbital and exhibit two ring currents with opposing directions, one being diatropic outside the molecular ring and the other paratropic inside (Figure S20).

In order to determine the ring-current strengths, the induced current density (*J*^ind^) is integrated over a square plane 6 Å from the center of the ring along the axis to one carbon, spanning 3 Å both above and below the plane of the ring. The ring-current strengths of Ca©B_8_C_4_^–^ (26.9 nA/T), Sr©BC_13_^+^ (47.5 nA/T), and Ba©B_3_C_12_^+^ (29.5 nA/T) exceed those of C_18_ (25.3 nA/T) and benzene (11.5 nA/T).^83^ In contrast, Ca©B_7_C_5_^–^ (18.6 nA/T), Ca©B_6_C_6_ (18.6 nA/T), and Ba©B_2_C_13_^–^ (18.2 nA/T) have weaker ring currents, while Sr©B_8_C_5_^–^ (−2.5 nA/T) and Ba©B_4_C_10_^–^ (−11.2 nA/T) show a net paratropic ring current strength due to a strong paratropic ring current contribution. The significant diatropic contribution from the alkaline-earth metal core is not considered in the estimation of the ring current strength. Indeed, the resulting values closely resemble those of the isolated dications: 40.6 nA/T for Ca^2+^, 18.2 nA/T for Sr^2+^, and 17.8 nA/T for Ba^2+^. In conjunction with the AdNDP analysis, these results support the notion of dual σ + π aromaticity. Still, there are two exceptions: Sr©B_8_C_5_^–^ and Ba©B_4_C_10_^–^, which could be characterized as antiaromatic. Intriguingly, these species achieve additional stabilization via σ + π delocalized bonds compared to their acyclic analogues.

### Energy Decomposition Analysis

The interactions between Ae and the ring can be further quantified from the results of the energy decomposition analysis (EDA)^84^ coupled with the natural orbital for chemical valence (NOCV)^85^ theory. We have explored five fragmentation schemes involving the Ae atom and the ring with different charge and electronic states (Tables S6–S10). The proposed guideline for selecting the appropriate fragmentation scheme is the magnitude of the orbital interaction energy (Δ*E*_orb_).^86^ Those fragments interacting with the smallest Δ*E*_orb_ are preferred. For the phAe clusters, Ae^2+^ in a singlet state participating in donor–acceptor interactions with singlet/doublet B_*x*_C_*y*_^*q*–2^ motifs gives the smallest Δ*E*_orb_ value. Table 1 presents the results of EDA-NOCV for Ca©B_8_C_4_^–^, and the corresponding results for the remaining cases are provided in Tables S11–S14. This analysis reveals that although, because of the chosen ionic fragmentation scheme, the Ae-ring interactions are predominantly electrostatic (66.0–82.9%) in nature, the stabilization originating from the covalent interaction is also substantial. The latter interaction accounts for 17.1–34.0% of the total attraction.

**Table 1:** EDA of Ca©B_8_C_4_^–^ Considering Ca and B_8_C_4_ with Ca^2+^ (S, 3p^6^4s^0^) + B_8_C_4_^3–^ (D) as Interacting Fragments at the M06-2X/TZ2P-ZORA Level^a^

energy	interaction	Ca^2+^ (S, 3p^6^4s^0^) + B_8_C_4_^3–^ (D)
Δ*E*_int_		–713.9
Δ*E*_Pauli_		67.9
Δ*E*_elstat_^b^		–620.9 (79.4%)
Δ*E*_orb_^b^		–160.9 (20.6%)
Δ*E*_orb(1)_^c^	B_8_C_4_^3–^ → Ca^2+^ (3d_σ_) σ-donation	–51.2 (31.8%)
Δ*E*_orb(2)_^c^	B_8_C_4_^3–^ → Ca^2+^ (3d_σ′_) σ-donation	–29.5 (18.3%)
Δ*E*_orb(3)_^c^	B_8_C_4_^3–^ → Ca^2+^ (4s) σ-donation	–17.8 (11.1%)
Δ*E*_orb(4)_^c^	B_8_C_4_^3–^ → Ca^2+^ (4p_σ_) σ-donation	–10.7 (6.7%)
Δ*E*_orb(5)_^c^	B_8_C_4_^3–^ → Ca^2+^ (4p_σ__′_) σ-donation	–10.7 (6.7%)
Δ*E*_orb(6)_^c^	B_8_C_4_^3–^ → Ca^2+^ (3d_π_) π-donation	–8.0 (5.0%)
Δ*E*_orb(7)_^c^	B_8_C_4_^3–^ → Ca^2+^ (3d_π′_) π-donation	–8.0 (5.0%)
Δ*E*_orb(rest)_^c^		–25.0 (15.5%)

a Energy values are given in kcal/mol.

b The percentage contribution with respect to total attraction is given in parentheses.

c The percentage contribution in parentheses is given with respect to total orbital interaction.

**Figure 4:**
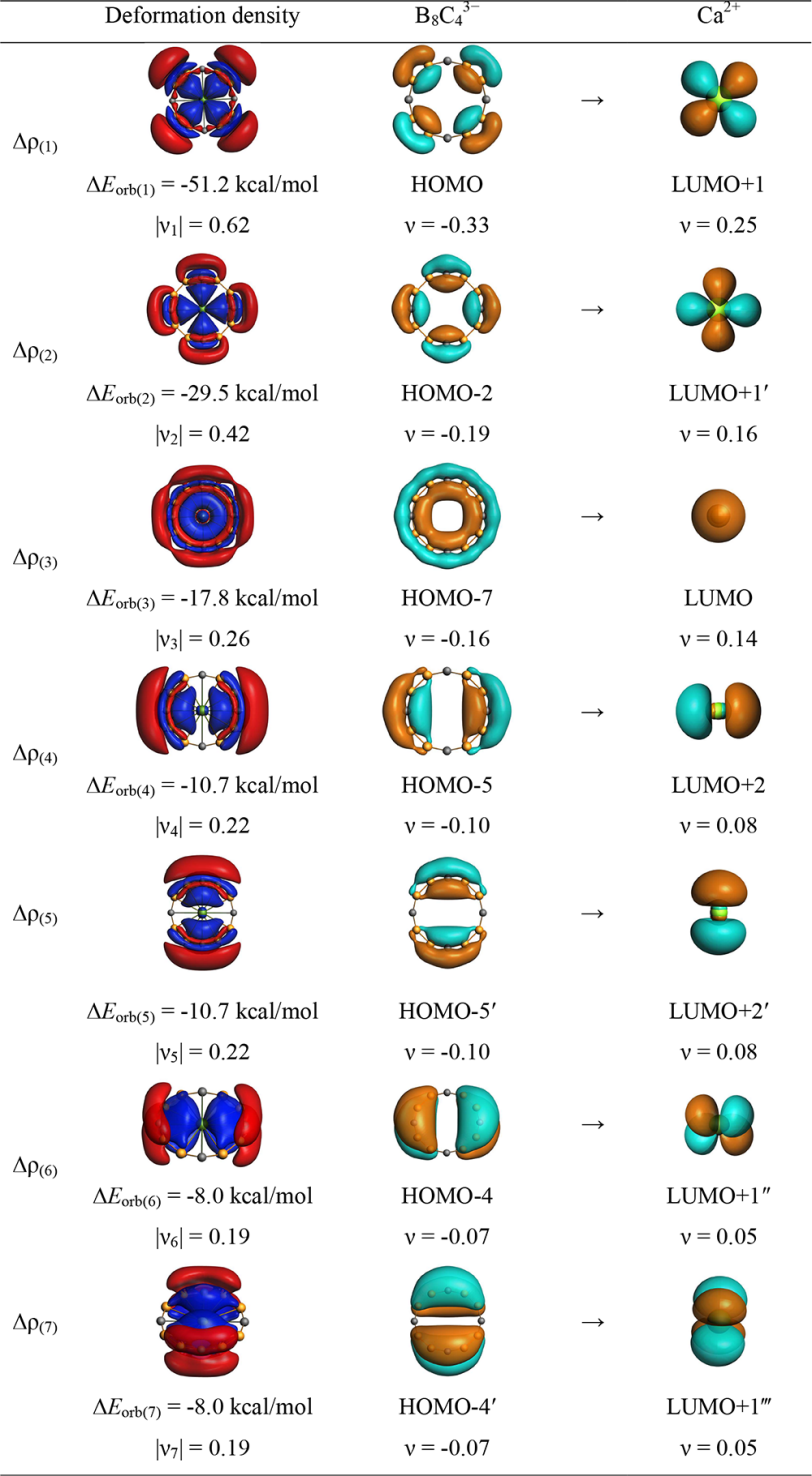
Plot of the deformation densities, Δρ_(1)–(7)_ shown as the sum of α and β electronic charge corresponding to Δ*E*_orb(1)–(7)_ and the related interacting orbitals of the fragments in Ca©B_8_C_4_^–^ at the M06-2X/TZ2P-ZORA level, taking Ca^2+^ (S, 3p^6^4s^0^) + B_8_C_4_^3–^ (D) as interacting fragments. The direction of the charge flow of the deformation densities is red → blue. The isovalue for Δρ_(1)–(2)_ is 0.0001 a.u., and the isovalue for Δρ_(3)–(7)_ is 0.00005.

Decomposing the total Δ*E*_orb_ into pairwise orbital interactions, Δ*E*_orb(*n*)_, provides valuable insights, particularly regarding the Ae atomic orbitals involved in the bonding. The deformation densities corresponding to Δ*E*_orb(*n*)_ and the interacting fragment orbitals, depicted in Figure 4 for Ca©B_8_C_4_^–^ and in Figures S25–S28 for others, support the idea of electron flow, where electron density shifts from the red to blue regions. There are seven distinct pairwise orbital interactions, Δ*E*_orb(1)–(7)_ isolated in all cases, which represent Ae^2+^ ← B_*y*_C_*x*_^*q*–2^ electron donation. The delocalized σ- and π-orbitals of B_*y*_C_*x*_ rings donate to the vacant s/p/d orbitals of Ae, especially to the d orbitals. For instance, in Ca©B_8_C_4_^–^, d orbitals account for 60% of the orbital interaction, with the remainder coming from s/p orbitals. Deformation densities, Δρ_(1)–(7)_ corresponding to Δ*E*_orb(1)–(7)_, depict the specific nature of charge transfer for Ca©B_8_C_4_^–^ (Figure 4). The most significant orbital interactions, Δ*E*_orb(1)_ and Δ*E*_orb(2)_, result from the delocalized σ-orbitals of B_8_C_4_^3–^ donating electrons to the d orbitals of Ca, constituting 50% of Δ*E*_orb_. Similar interaction patterns are noted in other representative examples, although not exhaustively detailed here. The involvement of high-lying f orbitals of Sr and Ba in the bonding is also found in the cases such as Sr©BC_13_^+^, Ba©B_4_C_10_^–^, and Ba©B_3_C_12_^+^ where the ring has a symmetric occupied orbital capable of donating electrons to the empty f orbital of Ae (see Figures S26–S28). Note that the participation of the f orbitals in the bonding produces 13.7–20.1% of the total covalent character in the respective cases.

Therefore, this quantitative analysis reveals that heavier Ae metals, namely, Ca, Sr, and Ba, extensively use their (*n* – 1)d atomic orbitals within the delocalized σ- and π-orbitals of B_*x*_C_*y*_ motifs, mirroring the behavior seen in transition metals within borometallic molecular structures. The unique capability of Ae d orbitals to participate enhances the stabilization of delocalized bonding, leading to double aromaticity and the stability of phAe clusters.

For Na©C_14_^+^ and Cs©C_18_^+^,^69^ considering Na^+^/Cs^+^ as one fragment and the carbon rings as another (see Tables S15 and S16), the Δ*E*_int_ values (−22.2 kcal/mol for Na and −14.9 kcal/mol for Cs) are significantly smaller than those computed for the present Ae cases. Surprisingly, the electrostatic interaction between Na^+^/Cs^+^ and the carbon ring is almost negligible. The stabilization primarily results from several weak orbital interaction terms. These data support the preference for heavier Ae elements over alkali metals in designing stable planar hypercoordinate systems.

## Conclusions

This work establishes a new record for the coordination number (15) in phAs. We achieved this by using mixed boron–carbon wheels, consisting of 12–15 atoms arranged in a ring structure, surrounding heavy alkaline-earth metals (Ca, Sr, and Ba). These metals, often referred to as “honorary transition metals”, have d orbitals crucial for stabilizing the phAe structures.

By applying a combination of geometrical and electronic criteria, we identified 180 potential stoichiometries to stabilize a phAe. Our focus on “double aromaticity” as a key stabilizing factor led us to found 10 clusters where a phAe structure emerges as the global minimum on their corresponding PESs (Ca©B_8_C_4_^–^, Ca©B_7_C_5_^–^, Ca©B_6_C_6_^–^, Ca©B_6_C_6_, Ca©C_13_, Sr©B_8_C_5_^–^, Sr©BC_13_^+^, Ba©B_4_C_10_^–^, Ba©B_3_C_12_^+^, and Ba©B_2_C_13_^+^). Chemical bonding analysis revealed the important contribution of the d orbitals from the Ae atoms in facilitating the delocalization of σ- and π-bonds within the ring system, further enhancing stability. Given that these structures are both global minima and likely kinetically stable, their detection in the gas phase might be achievable. The systematic design of these species opens a new avenue for predicting even more viable phAs and pave the way for novel 2D materials with unique properties.

## Supporting Information Available

Further computational details, structures of the low-energy isomers, dissociation energies, atomic charges, bonding distances, bond orders, AdNDP orbitals, analysis of ring current, EDA results, plot of deformation densities, and Cartesian coordinates of the title clusters (PDF)
