# Supplementary material for: Exploring the Use of “Honorary Transition Metals” To Push the Boundaries of Planar Hypercoordinate Alkaline-Earth Metals
Source: J Am Chem Soc. 2024 Jun 6;146(24):16689–97. doi: 10.1021/jacs.4c03977 (PMC11191695; doi:10.1021/jacs.4c03977)
Supplement: Supplementary file 1 — ja4c03977_si_001.pdf [file ja4c03977_si_001.pdf]

Supporting Information for

Exploring the Use of "Honorary Transition Metals" to Push the Boundaries of Planar Hypercoordinate Alkaline-Earth Metals

Xin-bo Liu,[a#] William Tiznado,[b#] Li-Juan Cui,[a] Jorge Barroso,[c] Luis Leyva-Parra[b] Lin-hong Miao,[a] Hui-yu Zhang,[a] Sudip Pan,*[,a] Gabriel Merino,*[,d] and Zhong-hua Cui*[a,e]

[a]*Institute of Atomic and Molecular Physics, Jilin University, Changchun 130023, China*

E-mail: sudip@jlu.edu.cn (SP); zcui@jlu.edu.cn (ZC)

[b] *Centro de Química Teórica & Computacional (CQT&C), Facultad de Ciencias Exactas, Departamento de Ciencias Químicas, Universidad Andrés Bello, Avenida República 275, 8370146 Santiago de Chile (Chile)*

[c] *Department of Chemistry, Clemson University, Clemson, South Carolina 29625, USA*

[d] *Departamento de Física Aplicada, Centro de Investigación y de Estudios Avanzados, Unidad Mérida. Km 6 Antigua Carretera a Progreso. Apdo. Postal 73, Cordemex, 97310, Mérida, Yuc., México.*

Email: gmerino@cinvestav.mx

[e] *Key Laboratory of Physics and Technology for Advanced Batteries (Ministry of Education), Jilin University, Changchun 130023, China*

[#]These authors contributed equally to this work.

Computational details

The initial structures of $AeB_xC_y^q$ clusters ($12 \leq x+y \leq 15$; q=0, ±1, $y > 0$) in both singlet and triplet spin states, as well as doublet and quartet spin states were optimized at the M06-2X/def2-SVP level.[1] Subsequent reminimization and characterization of low-lying energy isomers were conducted at the M06-2X/def2-TZVP level. The energies of these isomers were refined through single-point calculations at the CCSD(T)/def2-TZVP//M06-2X/def2-TZVP level,[2] incorporating the zero-point corrections calculated at the M06-2X level. For the lowest-energy structures, bonding analysis was done using different approaches, including natural bonding orbital (NBO),[3] electron localization function (ELF), adaptive natural density partitioning (AdDNP),[4] Bader analysis, and Laplacian of electron density. These analyses were donde using NBO7 and Multiwfn programs.[5] Aromaticity was assessed with the Gauge-Including Magnetically Induced Current (GIMIC) approach.[6] All these calculations were carried out using Gaussian 16.[7]

Energy Decomposition Analysis (EDA) and the Natural Orbital for Chemical Valence (NOCV) method were done using ADF at the M06-2X/TZ2P-ZORA//M06-2X/def2-TZVP level.[8] Finally, Born-Oppenheimer Molecular Dynamics (BOMD) simulations at 400K were conducted within the CP2K package.[9]

References

[1] Y. Zhao, D. G. Truhlar, *Theoretical Chemistry Accounts* **2008**, *120*, 215-241.

[2] J. A. Pople, M. Head-Gordon, K. Raghavachari, *The Journal of Chemical Physics* **1987**, *87*, 5968-5975.

[3] a) K. B. Wiberg, *Tetrahedron* **1968**, *24*, 1083-1096; b) J. P. Foster, F. Weinhold, *J. Am. Chem. Soc.* **1980**, *102*, 7211-7218; c) A. E. Reed, F. Weinhold, *The Journal of Chemical Physics* **1983**, *78*, 4066-4073; d) A. E. Reed, R. B. Weinstock, F. Weinhold, *The Journal of Chemical Physics* **1985**, *83*, 735-746.

[4] D. Y. Zubarev, A. I. Boldyrev, *Physical Chemistry Chemical Physics* **2008**, *10*, 5207.

[5] a) R. F. W. Bader, *Chemical Reviews* **1991**, *91*, 893-928; b) F. Cortés-Guzmán, R. F. W. Bader, *Coordination Chemistry Reviews* **2005**, *249*, 633-662; c) J. Poater, M. Duran, M. Solà, B. Silvi, *Chemical Reviews* **2005**, *105*, 3911-3947; d) T. Lu, F. Chen, *Journal of Computational Chemistry* **2011**, *33*, 580-592; e) E. D. Glendening, C. R. Landis, F. Weinhold, *J Comput Chem* **2019**, *40*, 2234-2241.

[6] a) J. Jusélius, D. Sundholm, J. Gauss, *The Journal of Chemical Physics* **2004**, *121*, 3952-3963; b) H. Fliegl, S. Taubert, O. Lehtonen, D. Sundholm, *Physical Chemistry Chemical Physics* **2011**, *13*, 20500.
[7] Gaussian 16, Revision C.01, M. J. Frisch, G. W. Trucks, H. B. Schlegel, G. E. Scuseria, M. A. Robb, J. R. Cheeseman, G. Scalmani, V. Barone, G. A. Petersson, H. Nakatsuji, X. Li, M. Caricato, A. V. Marenich, J. Bloino, B. G. Janesko, R. Gomperts, B. Mennucci, H. P. Hratchian, J. V. Ortiz, A. F. Izmaylov, J. L. Sonnenberg, Williams, F. Ding, F. Lipparini, F. Egidi, J. Goings, B. Peng, A. Petrone, T. Henderson, D. Ranasinghe, V. G. Zakrzewski, J. Gao, N. Rega, G. Zheng, W. Liang, M. Hada, M. Ehara, K. Toyota, R. Fukuda, J. Hasegawa, M. Ishida, T. Nakajima, Y. Honda, O. Kitao, H. Nakai, T. Vreven, K. Throssell, J. A. Montgomery Jr., J. E. Peralta, F. Ogliaro, M. J. Bearpark, J. J. Heyd, E. N. Brothers, K. N. Kudin, V. N. Staroverov, T. A. Keith, R. Kobayashi, J. Normand, K. Raghavachari, A. P. Rendell, J. C. Burant, S. S. Iyengar, J. Tomasi, M. Cossi, J. M. Millam, M. Klene, C. Adamo, R. Cammi, J. W. Ochterski, R. L. Martin, K. Morokuma, O. Farkas, J. B. Foresman, D. J. Fox, Gaussian. Inc., Wallingford, CT, **2019**.
[8] a) T. Ziegler, A. Rauk, *Theoretica Chimica Acta* **1977**, *46*, 1-10; b) M. Mitoraj, A. Michalak, *Organometallics* **2007**, *26*, 6576-6580; c) M. Mitoraj, A. Michalak, *Journal of Molecular Modeling* **2008**, *14*, 681-687; d) S. ADF2020, Theoretical Chemistry, Vrije Universiteit, Amsterdam, The Netherlands, http://www.scm.com.
[9] a) J. VandeVondele, M. Krack, F. Mohamed, M. Parrinello, T. Chassaing, J. Hutter, *Computer Physics Communications* **2005**, *167*, 103-128; b) T. D. Kühne, M. Iannuzzi, M. Del Ben, V. V. Rybkin, P. Seewald, F. Stein, T. Laino, R. Z. Khaliullin, O. Schütt, F. Schiffmann, D. Golze, J. Wilhelm, S. Chulkov, M. H. Bani-Hashemian, V. Weber, U. Borštnik, M. Taillefumier, A. S. Jakobovits, A. Lazzaro, H. Pabst, T. Müller, R. Schade, M. Guidon, S. Andermatt, N. Holmberg, G. K. Schenter, A. Hehn, A. Bussy, F. Belleflamme, G. Tabacchi, A. Glöß, M. Lass, I. Bethune, C. J. Mundy, C. Plessl, M. Watkins, J. VandeVondele, M. Krack, J. Hutter, *The Journal of Chemical Physics* **2020**, *152*, 194103.

Table S1. Summary of optimized $AeB_xC_y^q$ clusters (Ae= Ca, Sr, Ba; $x+y$=12, 13, 14, 15; q=0, ±1; $y > 0$) at the PBE0-D3/def2-TZVP and M06-2X/def2-TZVP levels. Color represent: Green for true minima, blue for saddle points with imaginary frequencies (out-of-plane mode at center Ae atom), and yellow for structures with twisted peripheral rings.

Systems	Ca		Sr		Ba	
	M06-2X	PBE0	M06-2X	PBE0	M06-2X	PBE0
**dodecacoordination**						
AeC_{12}^-	Blue	Blue	Blue	Blue	Blue	Blue
$AeB_4C_8^+$	Blue	Blue	Blue	Blue	Blue	Blue
$AeB_5C_7^+$	Blue	Blue	Blue	Blue	Blue	Blue
AeB_5C_7	Green	Green	Blue	Blue	Blue	Blue
$AeB_6C_6^+$	Blue	Blue	Blue	Blue	Blue	Blue
AeB_6C_6	Green	Green	Blue	Blue	Blue	Blue
$AeB_6C_6^-$	Green	Green	Blue	Blue	Blue	Blue
AeB_7C_5	Green	Green	Blue	Blue	Blue	Blue
$AeB_7C_5^-$	Green	Green	Blue	Blue	Blue	Blue
$AeB_8C_4^-$	Green	Green	Blue	Blue	Blue	Blue
**tridecacoordination**						
AeC_{13}^+	Blue	Blue	Blue	Blue	Blue	Blue
AeC_{13}	Green	Green	Blue	Blue	Blue	Blue
AeC_{13}^-	Yellow	Yellow	Blue	Blue	Blue	Blue
$AeBC_{12}$	Yellow	Yellow	Blue	Blue	Blue	Blue
$AeBC_{12}^-$	Yellow	Yellow	Blue	Green	Blue	Blue
$AeB_2C_{11}^-$	Yellow	Yellow	Blue	Blue	Blue	Blue
$AeB_6C_7^+$	Blue	Blue	Blue	Blue	Blue	Blue
$AeB_7C_6^+$	Green	Green	Blue	Green	Blue	Blue
AeB_7C_6	Yellow	Yellow	Blue	Blue	Blue	Blue
$AeB_8C_5^+$	Yellow	Yellow	Blue	Blue	Blue	Blue
AeB_8C_5	Yellow	Yellow	Green	Green	Blue	Blue
$AeB_8C_5^-$	Yellow	Yellow	Green	Green	Blue	Blue
AeB_9C_4	Green	Green	Green	Green	Blue	Blue
$AeB_9C_4^-$	Green	Green	Green	Green	Blue	Blue
$AeB_{10}C_3^-$	Green	Green	Green	Green	Blue	Blue
**tetradecacoordination**						
$AeBC_{13}^+$	Yellow	Yellow	Green	Green	Blue	Blue
$AeBC_{13}$	Yellow	Yellow	Yellow	Yellow	Blue	Blue
$AeB_2C_{12}^+$	Yellow	Yellow	Yellow	Yellow	Blue	Blue
AeB_2C_{12}	Yellow	Yellow	Yellow	Yellow	Blue	Blue
$AeB_2C_{12}^-$	Yellow	Yellow	Yellow	Yellow	Green	Green
AeB_3C_{11}	Yellow	Yellow	Yellow	Yellow	Blue	Blue
$AeB_3C_{11}^-$	Yellow	Yellow	Yellow	Yellow	Green	Green
$AeB_4C_{10}^-$	Yellow	Yellow	Yellow	Yellow	Green	Green
$AeB_8C_6^+$	Yellow	Yellow	Yellow	Yellow	Green	Blue
$AeB_9C_5^+$	Yellow	Yellow	Yellow	Yellow	Blue	Blue

AeB_9C_5						
$AeB_{10}C_4^+$						
$AeB_{10}C_4$						
$AeB_{10}C_4^-$						
$AeB_{11}C_3$						
$AeB_{11}C_3^-$						
$AeB_{12}C_2^-$						
pentadecacoordination						
$AeB_2C_{13}^+$						
$AeB_3C_{12}^+$						
AeB_3C_{12}						
$AeB_4C_{11}^+$						
AeB_4C_{11}						
$AeB_4C_{11}^-$						
AeB_5C_{10}						
$AeB_5C_{10}^-$						
$AeB_6C_9^-$						
$AeB_{10}C_5^+$						
$AeB_{11}C_4^+$						
$AeB_{11}C_4$						
$AeB_{12}C_3^+$						
$AeB_{12}C_3$						
$AeB_{12}C_3^-$						
$AeB_{13}C_2$						
$AeB_{13}C_2^-$						
$AeB_{14}C^-$						

Table S2. The summary of the global-minimum searches based on the target-orientated genetic algorithm. The green color represents the lowest-energy phAe candidates, whereas the lower non-phAe structures in energy were found as marked by red color at the M06-2X/def2-TZVP level.

Systems		Systems	
12 coordination		14 coordination	
CaB_5C_7		$SrBC_{13}^+$	
CaB_6C_6		$BaB_2C_{12}^-$	
$CaB_6C_6^-$		$BaB_3C_{11}^-$	
CaB_7C_5		$BaB_4C_{10}^-$	
$CaB_7C_5^-$		BaB_9C_5	
$CaB_8C_4^-$		$BaB_{10}C_4$	
		$BaB_{10}C_4^-$	
		$BaB_{11}C_3$	
		$BaB_{11}C_3^-$	
		$BaB_{12}C_2^-$	
13 coordination		15 coordination	
CaC_{13}		$BaB_2C_{13}^+$	
$CaB_7C_6^+$		$BaB_3C_{12}^+$	
CaB_9C_4		$BaB_5C_{10}^-$	
$CaB_9C_4^-$		$BaB_{12}C_3^-$	
$CaB_{10}C_3^-$		$BaB_{13}C_2^-$	
SrB_8C_5			
$SrB_8C_5^-$			
SrB_9C_4			
$SrB_9C_4^-$			
$SrB_{10}C_3^-$			

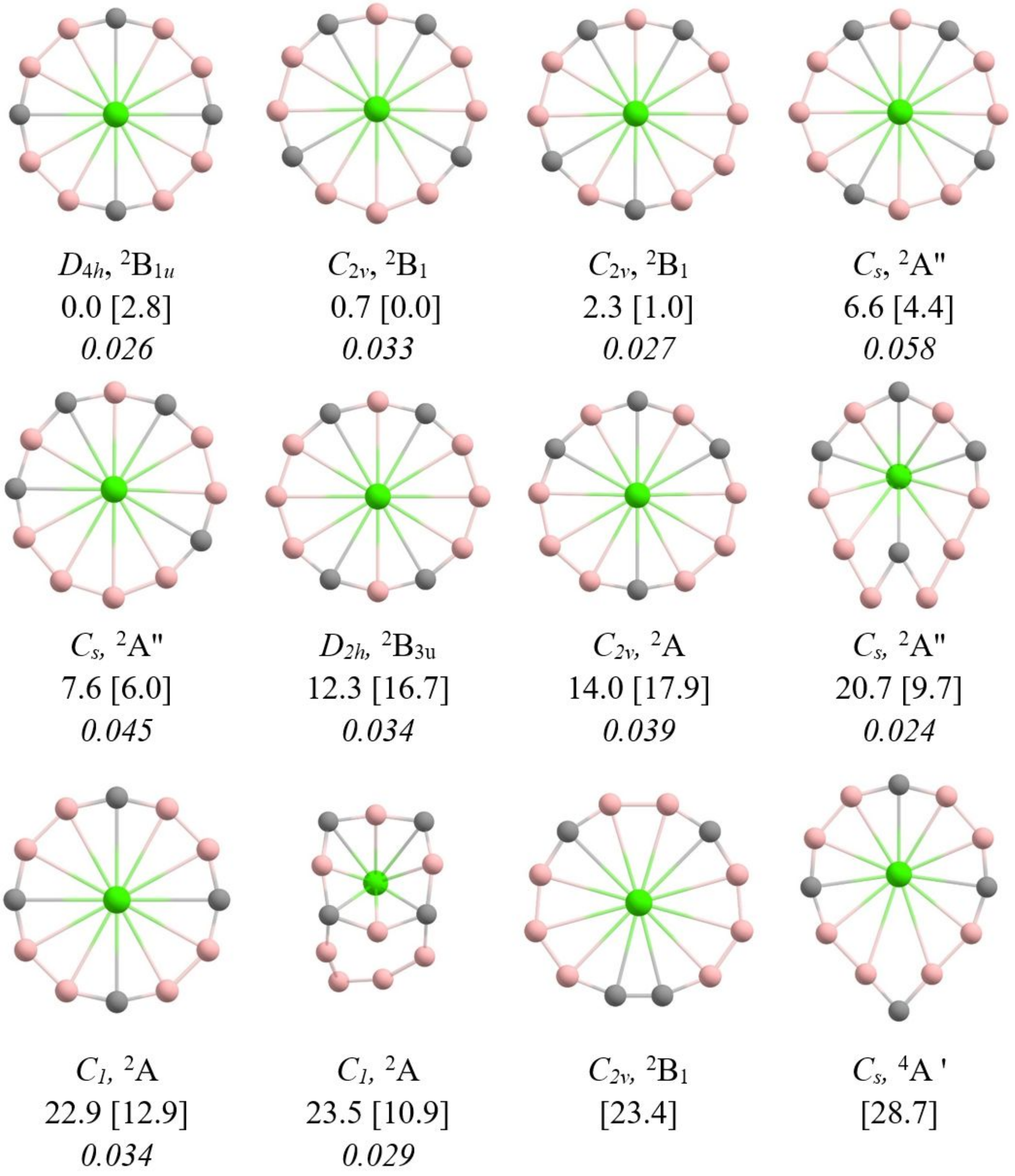

Figure S1. Structures of the low-energy isomers for $CaB_8C_4^-$. Relative energies in kcal/mol at both CCSD(T)/def2-TZVP//M06-2X/def2-TZVP and M06-2X/def2-TZVP (shown in square brackets) levels, including zero-point energy corrections at the M06-2X level. Point group symmetries, spectroscopic states, and T_1 diagnostic values (in italics). Color coding: Calcium in green, carbon in gray, and boron in pink.

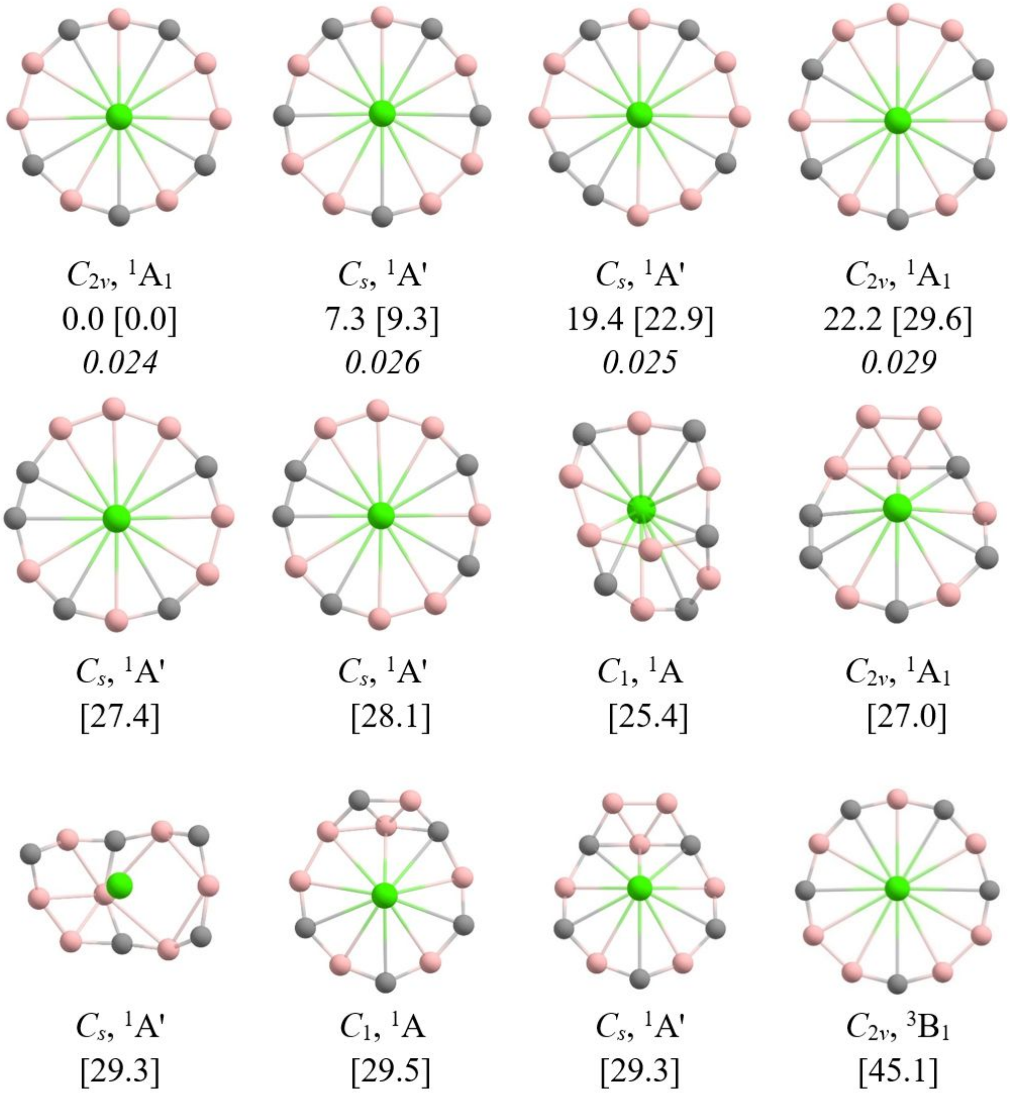

Figure S2. Structures of the low-energy isomers for $CaB_7C_5^-$. Relative energies in kcal/mol at both CCSD(T)/def2-TZVP//M06-2X/def2-TZVP and M06-2X/def2-TZVP (shown in square brackets) levels, including zero-point energy corrections at the M06-2X level. Point group symmetries, spectroscopic states, and T_1 diagnostic values (in italics). Color coding: Calcium in green, carbon in gray, and boron in pink.

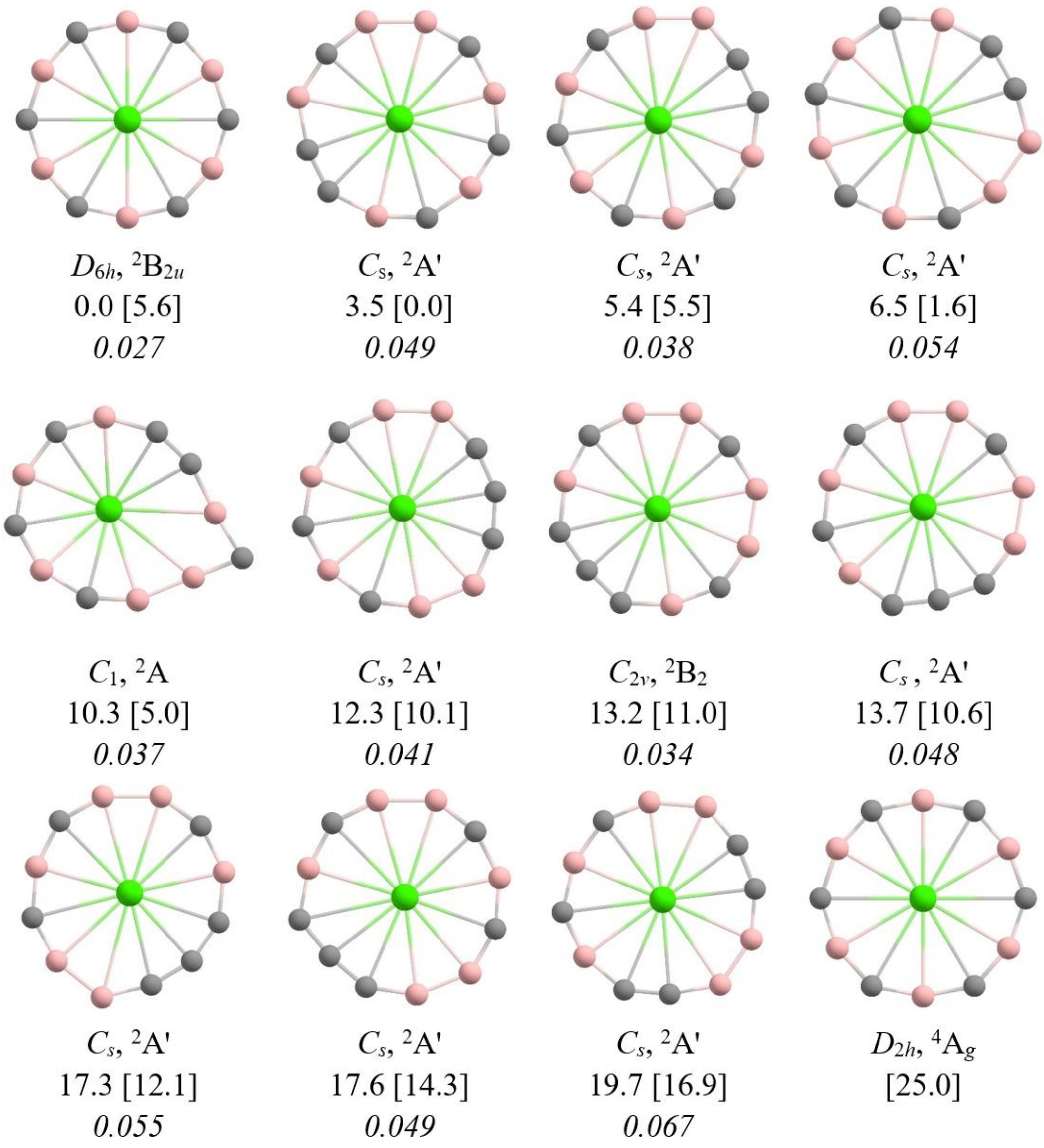

Figure S3. Structures of the low-energy isomers for $CaB_6C_6^-$. Relative energies in kcal/mol at both CCSD(T)/def2-TZVP//M06-2X/def2-TZVP and M06-2X/def2-TZVP (shown in square brackets) levels, including zero-point energy corrections at the M06-2X level. Point group symmetries, spectroscopic states, and T_1 diagnostic values (in italics). Color coding: Calcium in green, carbon in gray, and boron in pink.

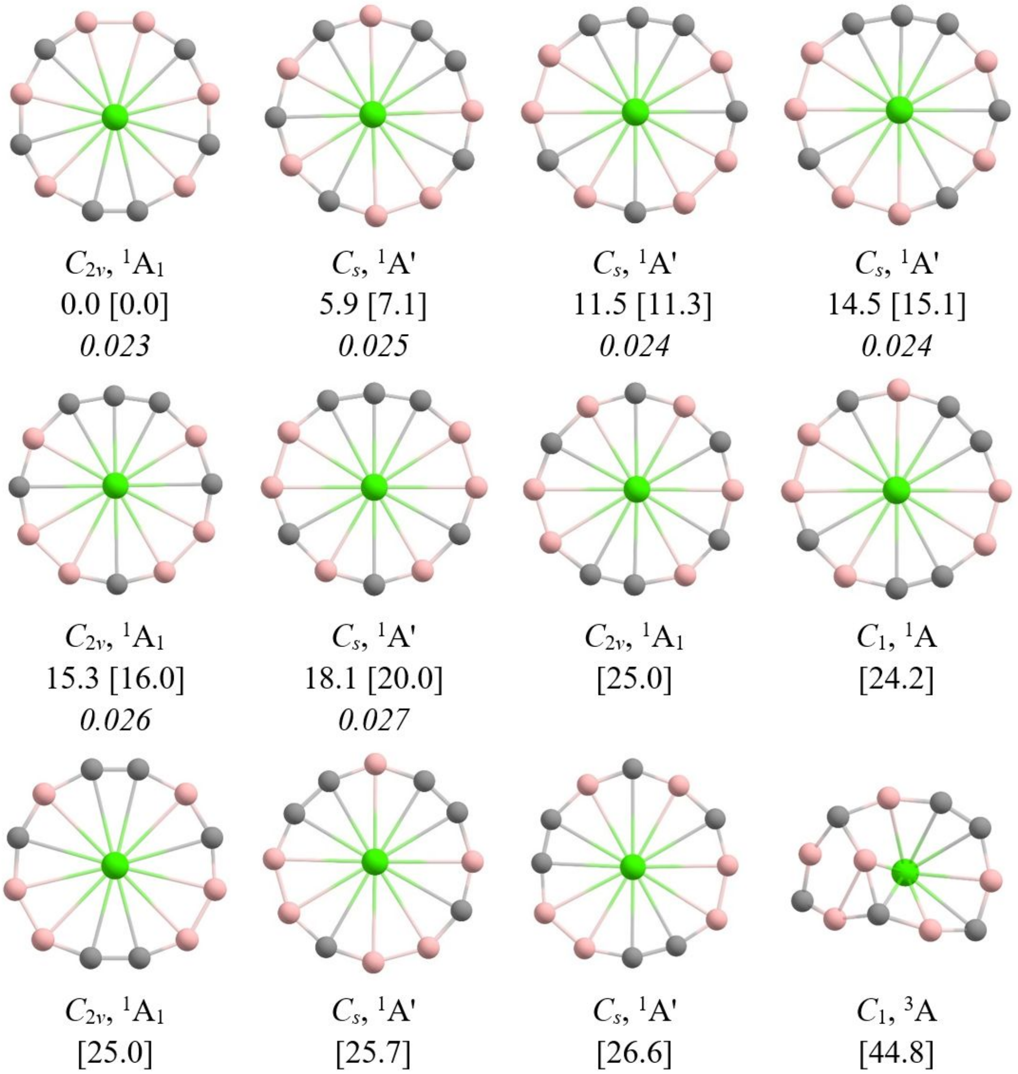

Figure S4. Structures of the low-energy isomers for CaB_6C_6. Relative energies in kcal/mol at both CCSD(T)/def2-TZVP//M06-2X/def2-TZVP and M06-2X/def2-TZVP (shown in square brackets) levels, including zero-point energy corrections at the M06-2X level. Point group symmetries, spectroscopic states, and T_1 diagnostic values (in italics). Color coding: Calcium in green, carbon in gray, and boron in pink.

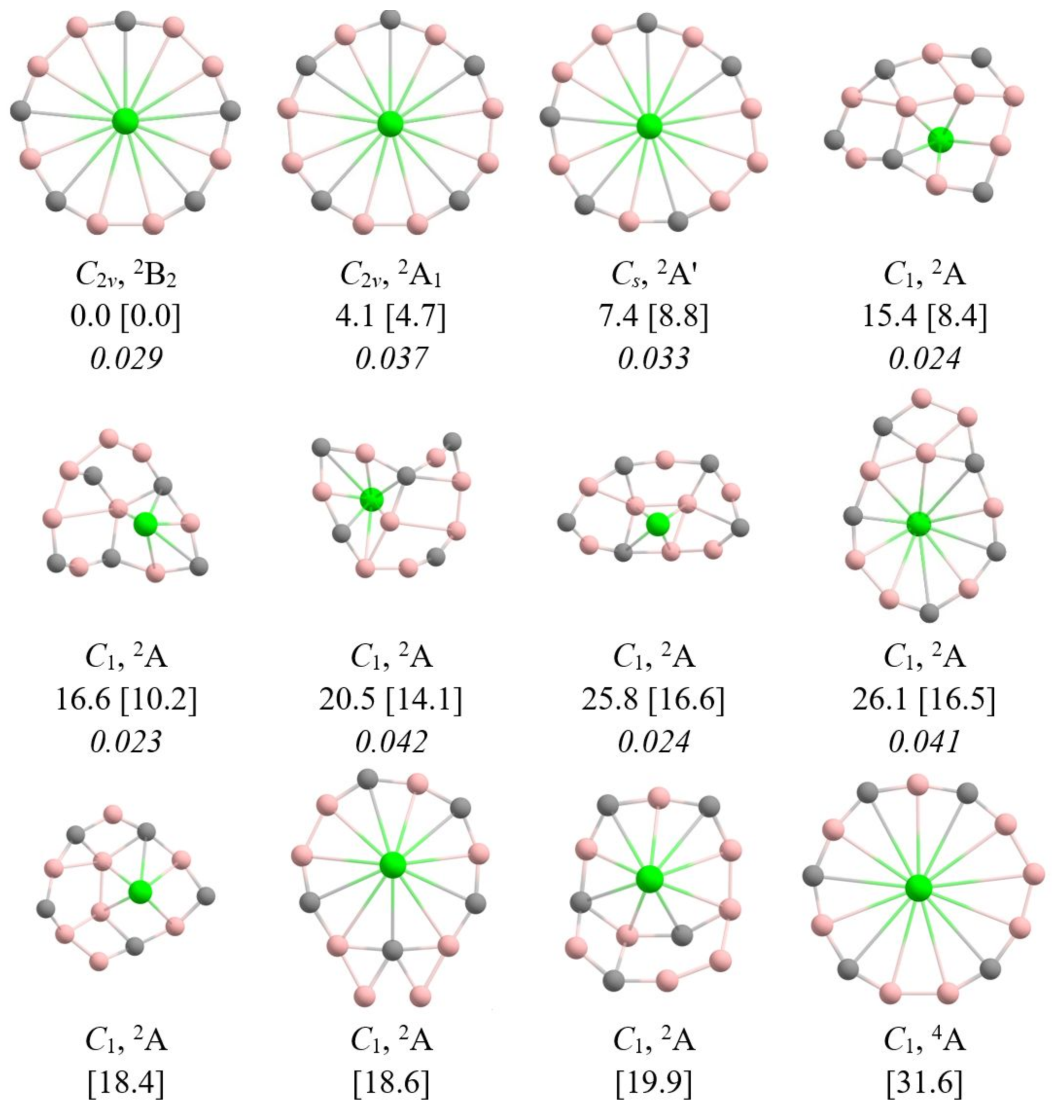

Figure S5. Structures of the low-energy isomers for $SrB_8C_5^-$. Relative energies in kcal/mol at both CCSD(T)/def2-TZVP//M06-2X/def2-TZVP and M06-2X/def2-TZVP (shown in square brackets) levels, including zero-point energy corrections at the M06-2X level. Point group symmetries, spectroscopic states, and T_1 diagnostic values (in italics). Color coding: Strontium in green, carbon in gray, and boron in pink.

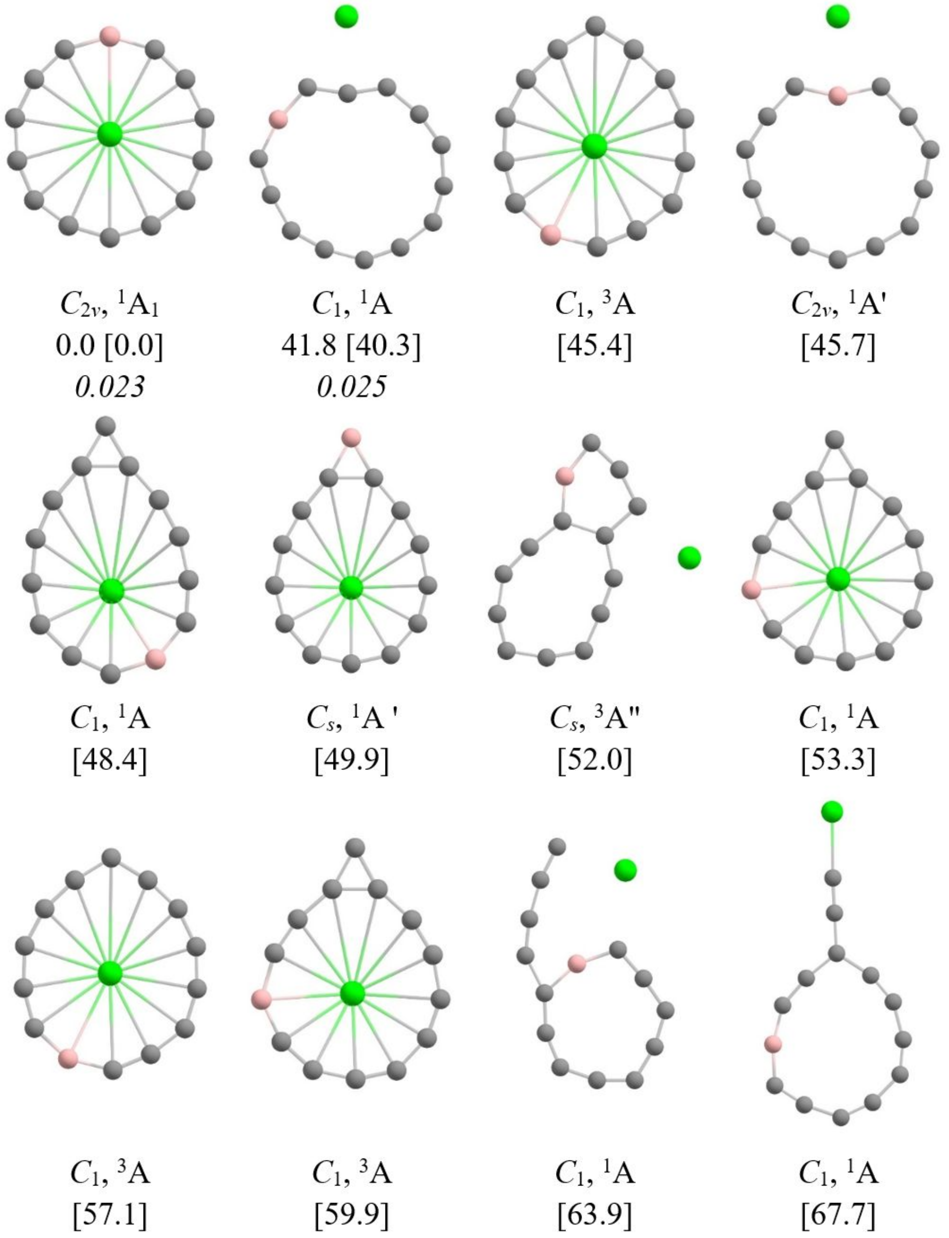

Figure S6. Structures of the low-energy isomers for $SrBC_{13}^{+}$. Relative energies in kcal/mol at both CCSD(T)/def2-TZVP//M06-2X/def2-TZVP and M06-2X/def2-TZVP (shown in square brackets) levels, including zero-point energy corrections at the M06-2X level. Point group symmetries, spectroscopic states, and T_1 diagnostic values (in italics). Color coding: Strontium in green, carbon in gray, and boron in pink.

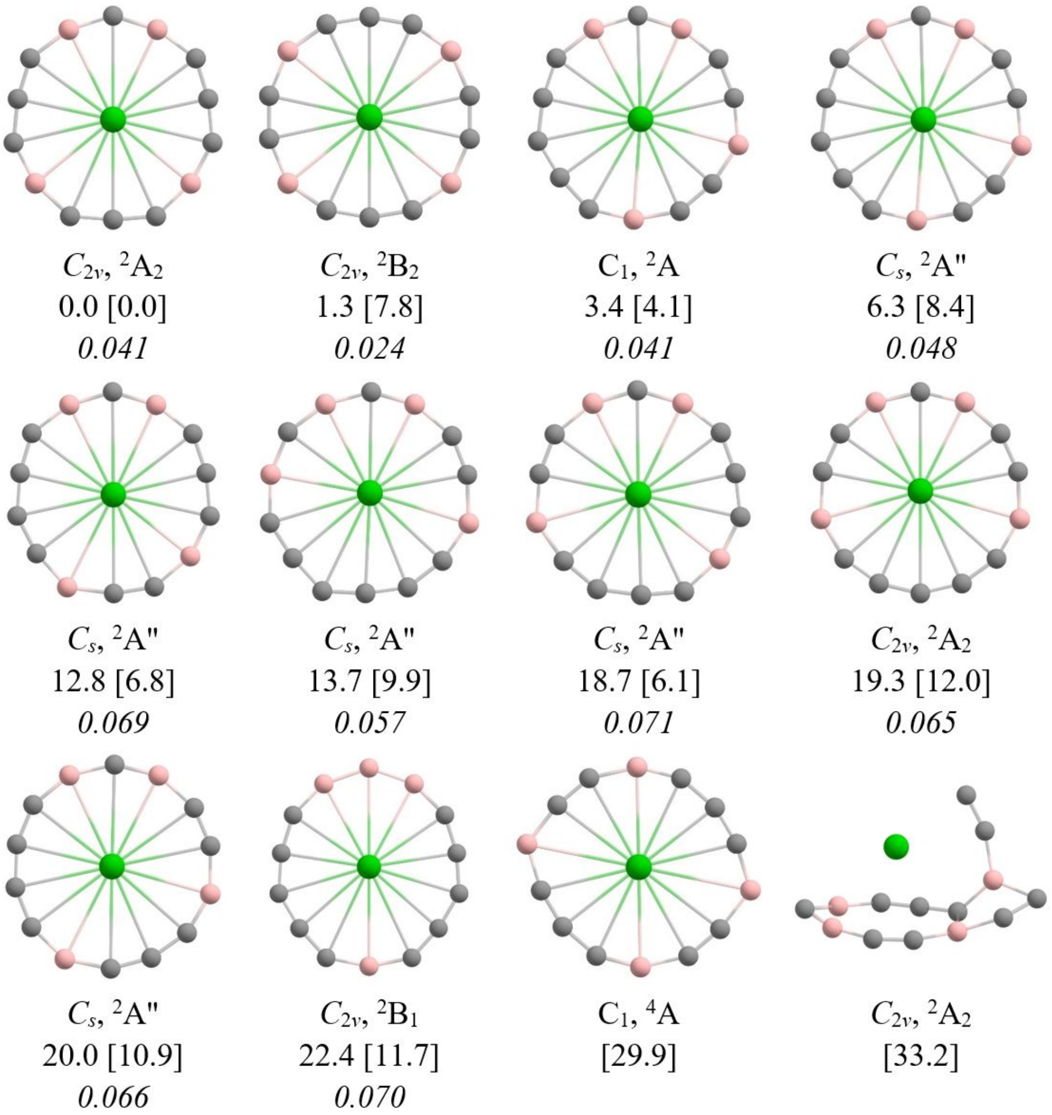

Figure S7. Structures of the low-energy isomers for $BaB_4C_{10}^-$. Relative energies in kcal/mol at both CCSD(T)/def2-TZVP//M06-2X/def2-TZVP and M06-2X/def2-TZVP (shown in square brackets) levels, including zero-point energy corrections at the M06-2X level. Point group symmetries, spectroscopic states, and T_1 diagnostic values (in italics). Color coding: Barium in green, carbon in gray, and boron in pink.

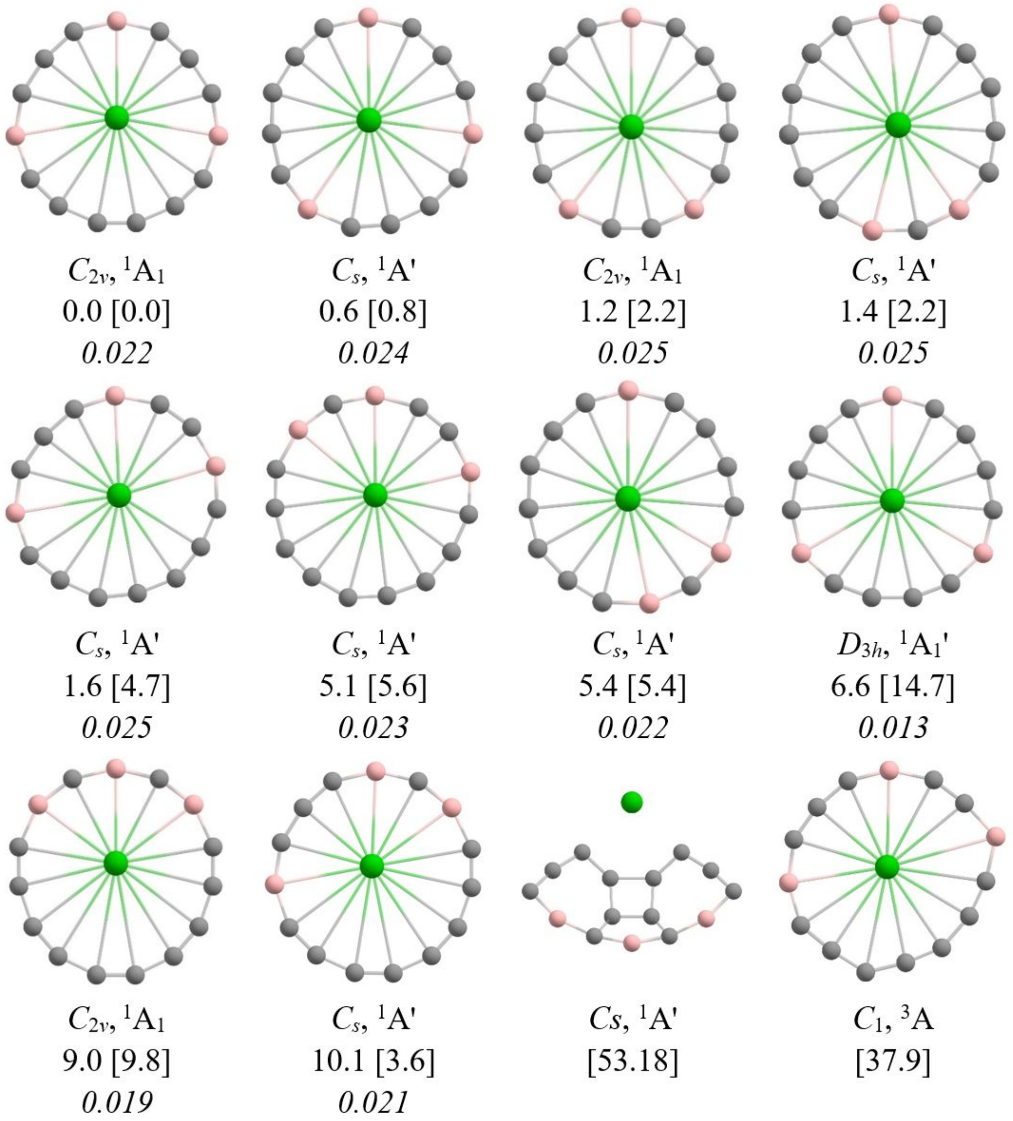

Figure S8. Structures of the low-energy isomers for $BaB_3C_{12}^+$. Relative energies in kcal/mol at both CCSD(T)/def2-TZVP//M06-2X/def2-TZVP and M06-2X/def2-TZVP (shown in square brackets) levels, including zero-point energy corrections at the M06-2X level. Point group symmetries, spectroscopic states, and T_1 diagnostic values (in italics). Color coding: Barium in green, carbon in gray, and boron in pink.

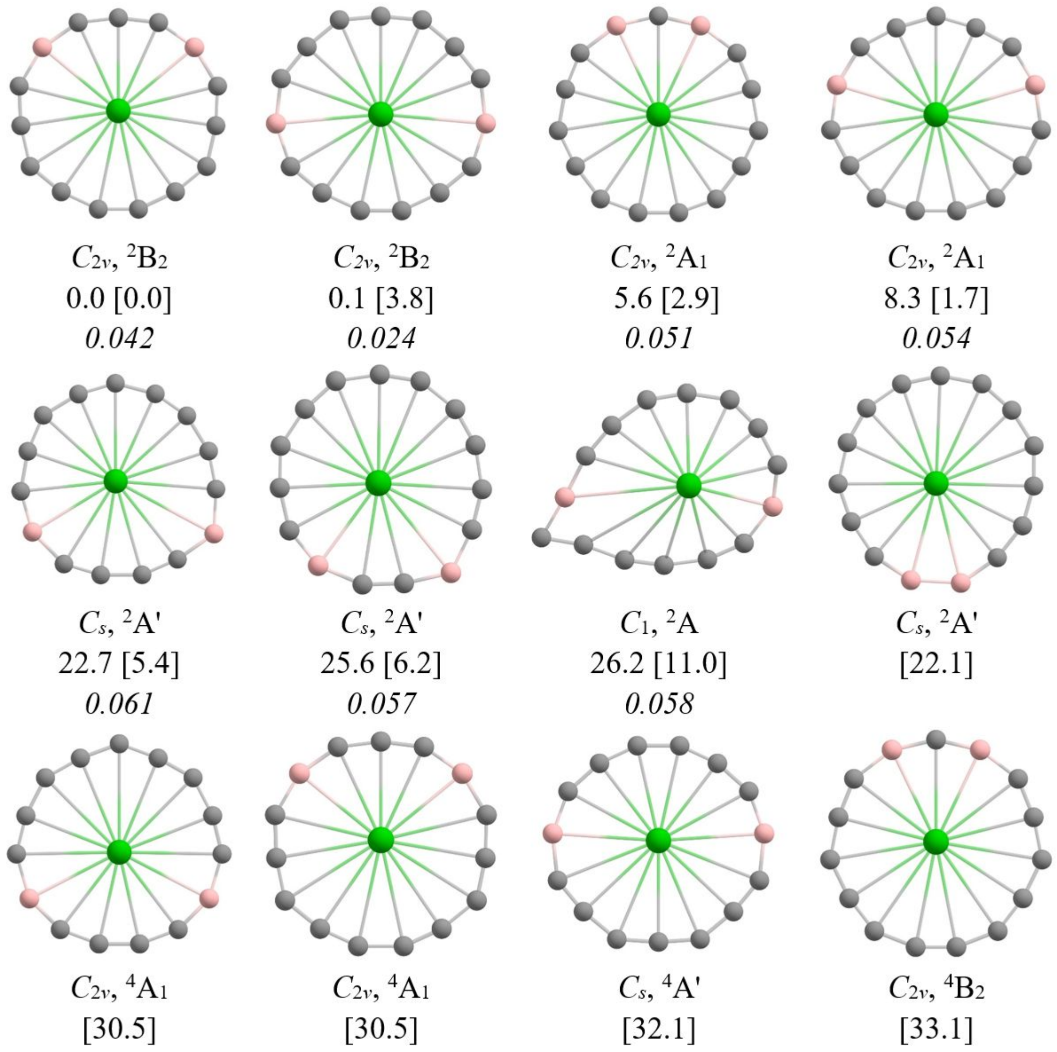

Figure S9. Structures of the low-energy isomers for $BaB_2C_{13}^+$. Relative energies in kcal/mol at both CCSD(T)/def2-TZVP//M06-2X/def2-TZVP and M06-2X/def2-TZVP (shown in square brackets) levels, including zero-point energy corrections at the M06-2X level. Point group symmetries, spectroscopic states, and T_1 diagnostic values (in italics). Color coding: Barium in green, carbon in gray, and boron in pink.

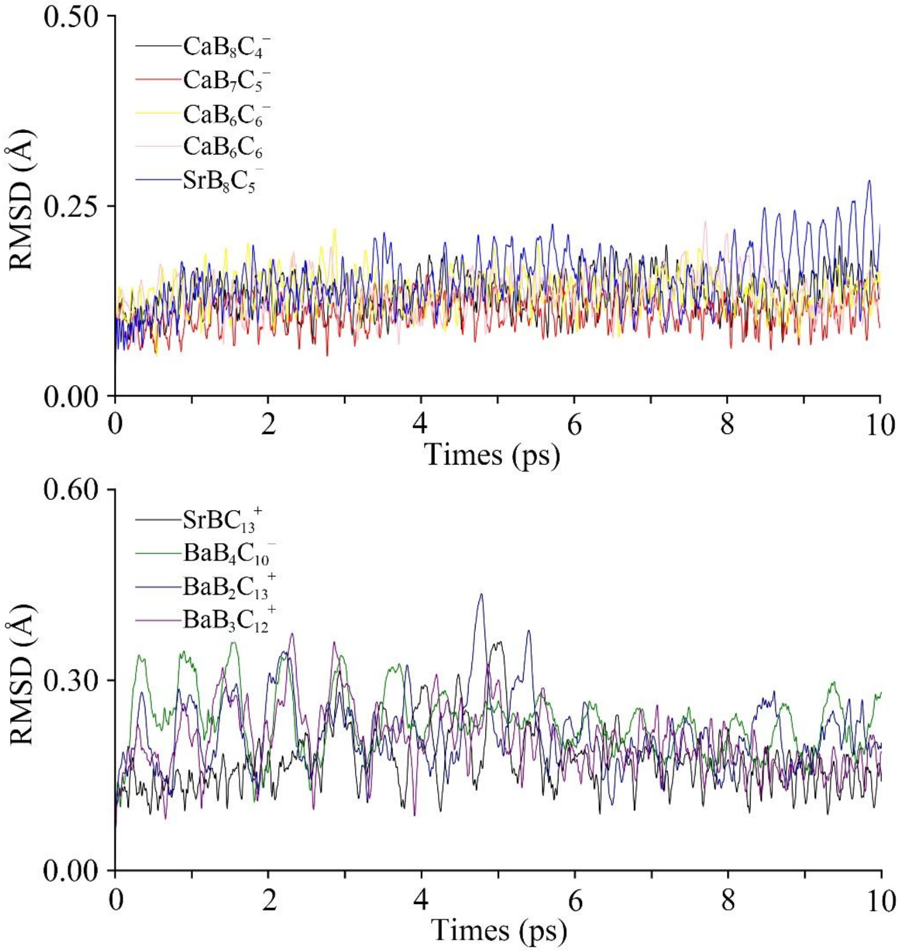

Figure S10. Root mean square deviation (RMSD) versus simulation time for phAe computed at the PBE/DZVP-MOLOPT-SR-GTH level.

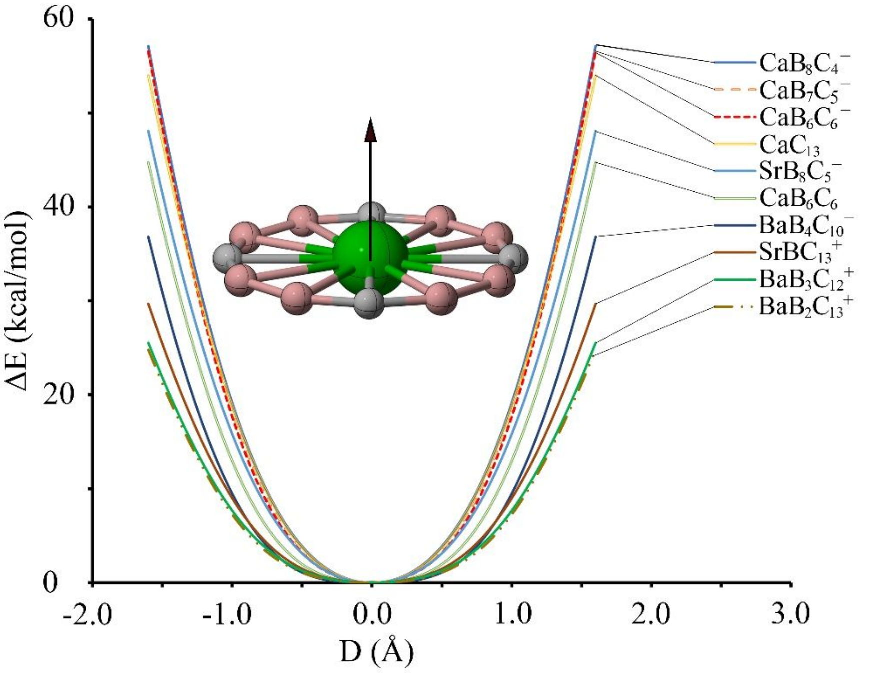

Figure S11. Computed potential energy curves for out-of-plane displacement of the central phAe atom at the M06-2X/def2-TZVP level.

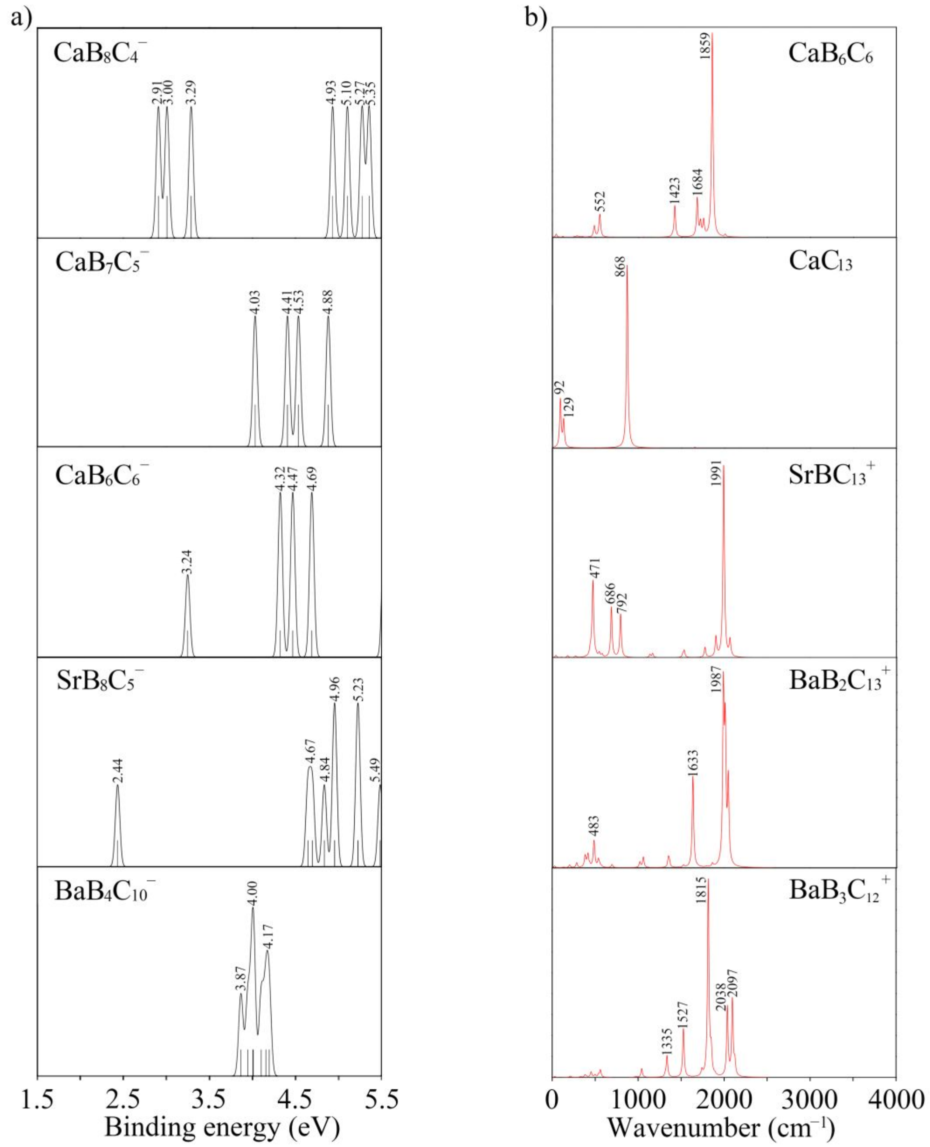

Figure S12. Simulated a) photoelectron spectrum (PES), b) infrared spectrums (IR) of phAe clusters.

Table S3. Dissociation energy (ΔE_{diss}) in kcal/mol of three dissociation pathways leading to Ae and B_xC_y fragments determined at the M06-2X/def2-TZVP level.

Pathway	ΔE_{dissoc}
	$Ca©B_8C_4^-$
$Ca©B_8C_4^- \rightarrow Ca + B_8C_4^-$	111.5
$Ca©B_8C_4^- \rightarrow Ca^+ + B_8C_4^{2-}$	301.2
$Ca©B_8C_4^- \rightarrow Ca^{2+} + B_8C_4^{3-}$	707.2
	$Ca©B_7C_5^-$
$Ca©B_7C_5^- \rightarrow Ca + B_7C_5^-$	193.6
$Ca©B_7C_5^- \rightarrow Ca^+ + B_7C_5^{2-}$	317.2
$Ca©B_7C_5^- \rightarrow Ca^{2+} + B_7C_5^{3-}$	683.9
	$Ca©B_6C_6^-$
$Ca©B_6C_6^- \rightarrow Ca + B_6C_6^-$	177.3
$Ca©B_6C_6^- \rightarrow Ca^+ + B_6C_6^{2-}$	322.7
$Ca©B_6C_6^- \rightarrow Ca^{2+} + B_6C_6^{3-}$	711.8
	$Ca©B_6C_6$
$Ca©B_6C_6 \rightarrow Ca + B_6C_6$	193.4
$Ca©B_6C_6 \rightarrow Ca^+ + B_6C_6^-$	224.9
$Ca©B_6C_6 \rightarrow Ca^{2+} + B_6C_6^{2-}$	496.4
	$Ca©C_{13}$
$Ca©C_{13} \rightarrow Ca + C_{13}$	138.0
$Ca©C_{13} \rightarrow Ca^+ + C_{13}^-$	204.7
$Ca©C_{13} \rightarrow Ca^{2+} + C_{13}^{2-}$	535.4
	$Sr©B_8C_5^-$
$Sr©B_8C_5^- \rightarrow Sr + B_8C_5^-$	134.2
$Sr©B_8C_5^- \rightarrow Sr^+ + B_8C_5^{2-}$	256.2
$Sr©B_8C_5^- \rightarrow Sr^{2+} + B_8C_5^{3-}$	645.1
	$Sr©BC_{13}^+$
$Sr©BC_{13}^+ \rightarrow Sr + BC_{13}^+$	155.2
$Sr©BC_{13}^+ \rightarrow Sr^+ + BC_{13}$	106.3
$Sr©BC_{13}^+ \rightarrow Sr^{2+} + BC_{13}^-$	269.2
	$Ba©B_4C_{10}^-$
$Ba©B_4C_{10}^- \rightarrow Ba + B_4C_{10}^-$	161.6
$Ba©B_4C_{10}^- \rightarrow Ba^+ + B_4C_{10}^{2-}$	283.7
$Ba©B_4C_{10}^- \rightarrow Ba^{2+} + B_4C_{10}^{3-}$	603.5
	$Ba©B_3C_{12}^+$
$Ba©B_3C_{12}^+ \rightarrow Ba + B_3C_{12}^+$	187.5
$Ba©B_3C_{12}^+ \rightarrow Ba^+ + B_3C_{12}$	121.8
$Ba©B_2C_{12}^+ \rightarrow Ba^{2+} + B_3C_{12}^-$	258.9
	$Ba©B_2C_{13}^+$
$Ba©B_2C_{13}^+ \rightarrow Ba + B_2C_{13}^+$	142.9
$Ba©B_2C_{13}^+ \rightarrow Ba^+ + B_2C_{13}$	79.5
$Ba©B_2C_{13}^+ \rightarrow Ba^{2+} + B_2C_{13}^-$	248.8

$Na©C_{14}^{+}$	
$Na©C_{14}^{+} \rightarrow Na + C_{14}^{+}$	101.9
$Na©C_{14}^{+} \rightarrow Na^{+} + C_{14}$	22.7
$Cs©C_{18}^{+}$	
$Cs©C_{18}^{+} \rightarrow Cs + C_{18}^{+}$	119.8
$Cs©C_{18}^{+} \rightarrow Cs^{+} + C_{18}$	15.2

Table S4. The various atomic charges of the lowest-energy phAe structures were computed at the M06-2X/def2-TZVP level. The Q^{max} and Q^{min} values refer to the largest and smallest atomic charge in |e| of B and C atoms. The NPA/CM5/Hirshfeld/QTAIM/Voronoi atomic charges are given in order with "/".

System	$Ca©B_8C_4^-$	$Ca©B_7C_5^-$
Q_{Ae}	1.83/1.07/0.49/1.53/0.47	1.85/1.13/0.52/1.56/0.51
Q_B^{max}	0.14/-0.21/-0.05/0.90/-0.05	0.60/-0.19/0.06/1.90/0.11
Q_B^{min}	0.14/-0.21/-0.05/0.90/-0.05	0.12/-0.23/-0.06/1.02/-0.06
Q_C^{max}	-0.98/-0.09/-0.27/-2.37/-0.27	-1.01/-0.12/-0.29/-2.40/-0.31
Q_C^{min}	-0.98/-0.09/-0.27/-2.37/-0.27	-1.07/-0.15/-0.30/-2.45/-0.36

Sys	$Ca©B_6C_6^-$	$Ca©B_6C_6$
Q_{Ae}	1.85/1.17/0.53/1.56/0.50	1.84/1.18/0.58/1.57/0.52
Q_B^{max}	0.38/-0.27/0.00/1.83/0.16	0.66/-0.13/0.10/1.05/0.06
Q_B^{min}	0.38/-0.27/0.00/1.83/0.16	0.28/-0.16/0.04/1.84/0.10
Q_C^{max}	-0.85/-0.09/-0.25/-2.26/	-0.32/0.00/-0.06/-0.88/-0.04
Q_C^{min}	-0.85/-0.09/-0.25/-2.26/	-1.03/-0.08/-0.25/-2.38/-0.28

Sys	$Ca©C_{13}$	$Sr©B_8C_5^-$
Q_{Ae}	1.90/1.09/0.74/1.70/0.63	1.88/1.18/0.63/1.59/0.54
Q_B^{max}		0.65/-0.18/0.07/1.92/0.13
Q_B^{min}		0.18/-0.20/-0.05/0.91/-0.05
Q_C^{max}	-0.09/-0.08/-0.06/-0.13/-0.06	-1.07/-0.12/-0.29/-2.39/-0.29
Q_C^{min}	-0.20/-0.08/-0.06/-0.13/-0.06	-1.08/-0.13/-0.31/-2.46/-0.35

Sys	$Sr©BC_{13}^{+}$	$Ba©B_4C_{10}^-$
Q_{Ae}	1.91/1.24/0.87/1.72/0.62	1.90/1.25/0.68/1.64/0.45
Q_B^{max}	0.53/-0.10/0.16/1.89/0.17	0.55/-0.23/0.04/1.89/0.10
Q_B^{min}	0.53/-0.10/0.16/1.89/0.17	0.39/-0.27/0.01/1.84/0.05
Q_C^{max}	0.02/0.02/0.03/-0.10/0.07	0.02/-0.06/-0.04/-0.41/0.04

Q_C^{min}	-0.44/-0.04/-0.09/-0.01/-0.01	-1.14/-0.17/-0.34/-2.50/-0.38

Sys	Ba©$B_3C_{12}^+$	Ba©$B_2C_{13}^+$
Q_{Ae}	1.90/1.26/0.88/1.71/0.55	1.91/1.26/0.89/1.72/0.54
Q_B^{max}	0.65/-0.08/0.19/1.96/0.23	0.64/-0.07/0.19/1.91/0.12
Q_B^{min}	0.57/-0.08/0.16/1.91/0.18	0.64/-0.07/0.19/1.91/0.12
Q_C^{max}	0.10/0.03/0.03/-0.30/0.12	0.12/0.03/0.05/-0.41/0.06
Q_C^{min}	-0.59/-0.06/-0.15/-1.12/-0.15	-0.59/-0.06/-0.13/-1.16/-0.29

Table S5. Bonding distances (in Å), and Mayer bond order (BO) for the lowest-energy phAe structures determined at the M06-2X/def2-TZVP level. B^{max} and B^{min} denotes the largest and smallest bond lengths, while BO^{max}, and BO^{min} represent the highest and lowest bond orders for C-C, B-C, Ae-B, and Ae-C bonds, respectively.

System	Ca©$B_8C_4^-$	Ca©$B_7C_5^-$	Ca©$B_6C_6^-$	Ca©B_6C_6	Ca©C_{13}
$B_{Ae\text{-}B}^{max}$	2.798	2.795	2.664	2.771	
$B_{Ae\text{-}B}^{min}$	2.798	2.665	2.664	2.663	
$B_{Ae\text{-}C}^{max}$	2.749	2.772	2.740	2.704	2.695
$B_{Ae\text{-}C}^{min}$	2.749	2.721	2.740	2.685	2.695
$B_{B\text{-}B}^{max}$	1.553	1.592		1.595	
$B_{B\text{-}B}^{min}$	1.553	1.592		1.595	
$B_{B\text{-}C}^{max}$	1.385	1.431	1.401	1.467	
$B_{B\text{-}C}^{min}$	1.385	1.352	1.401	1.351	
$B_{C\text{-}C}^{max}$				1.235	1.290
$B_{C\text{-}C}^{min}$				1.235	1.290
$BO_{Ae\text{-}B}^{max}$	0.15	0.13	0.13	0.14	
$BO_{Ae\text{-}B}^{min}$	0.15	0.12	0.13	0.11	
$BO_{Ae\text{-}C}^{max}$	0.14	0.15	0.13	0.17	0.08
$BO_{Ae\text{-}C}^{min}$	0.14	0.13	0.13	0.11	0.08
$BO_{B\text{-}B}^{max}$	1.37	1.23		1.19	
$BO_{B\text{-}B}^{min}$	1.37	1.23		1.19	
$BO_{B\text{-}C}^{max}$	1.65	1.94	1.65	1.96	
$BO_{B\text{-}C}^{min}$	1.65	1.44	1.65	1.29	
$BO_{C\text{-}C}^{max}$				2.25	1.79
$BO_{C\text{-}C}^{min}$				2.25	1.79

System	Sr©$B_8C_5^-$	Sr©BC_{13}^+	Ba©$B_4C_{10}^-$	Ba©$B_3C_{12}^+$	Ba©$B_2C_{13}^+$
$B_{Ae\text{-}B}^{max}$	3.049	2.929	3.004	3.262	3.134
$B_{Ae\text{-}B}^{min}$	2.921	2.929	2.995	3.053	3.134
$B_{Ae\text{-}C}^{max}$	3.029	3.093	3.163	3.372	3.301
$B_{Ae\text{-}C}^{min}$	2.862	2.791	2.910	2.952	2.936
$B_{B\text{-}B}^{max}$	1.602				
$B_{B\text{-}B}^{min}$	1.585				
$B_{B\text{-}C}^{max}$	1.417	1.399	1.439	1.448	1.426
$B_{B\text{-}C}^{min}$	1.350	1.399	1.378	1.355	1.379
$B_{C\text{-}C}^{max}$		1.298	1.316	1.341	1.321
$B_{C\text{-}C}^{min}$		1.269	1.267	1.228	1.250
$BO_{Ae\text{-}B}^{max}$	0.13	0.06	0.09	0.06	0.05
$BO_{Ae\text{-}B}^{min}$	0.08	0.06	0.08	0.03	0.05
$BO_{Ae\text{-}C}^{max}$	0.16	0.13	0.12	0.10	0.10
$BO_{Ae\text{-}C}^{min}$	0.13	0.05	0.01	0.03	0.05
$BO_{B\text{-}B}^{max}$	1.19				
$BO_{B\text{-}B}^{min}$	1.19				

BO_{B-C}^{max}	1.92	1.69	1.99	2.02	1.82
BO_{B-C}^{min}	1.49	1.69	1.56	1.43	1.53
BO_{C-C}^{max}		1.83	1.86	2.40	2.05
BO_{C-C}^{min}		1.60	1.44	1.37	1.38

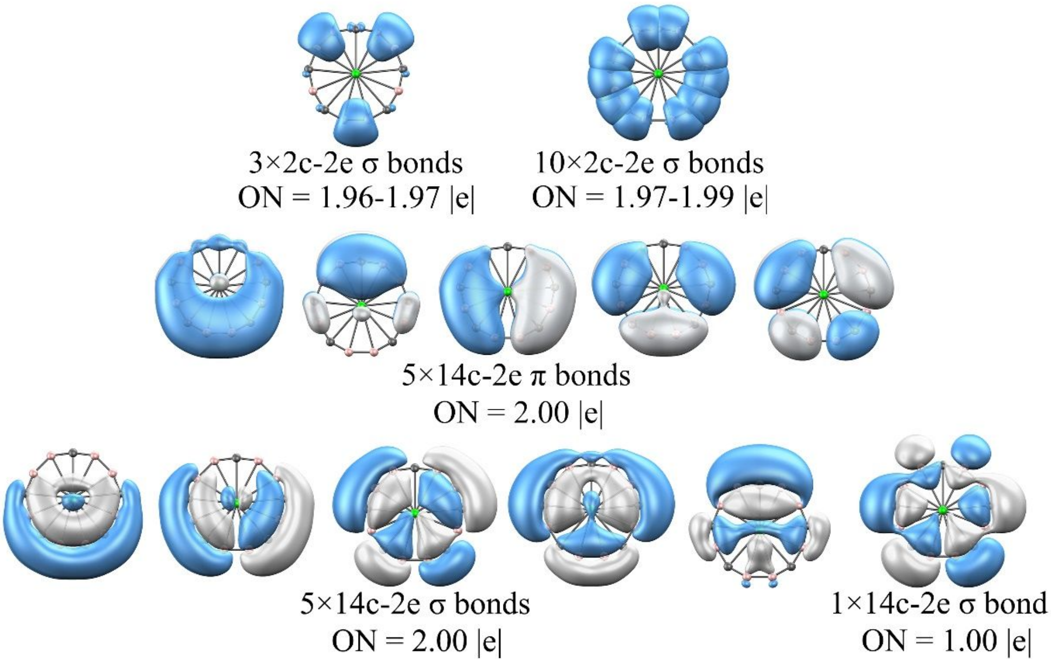

Figure S13. AdNDP orbitals of Sr©$B_8C_5^-$ at the M06-2X/def2-TZVP level. ON denotes the occupation number.

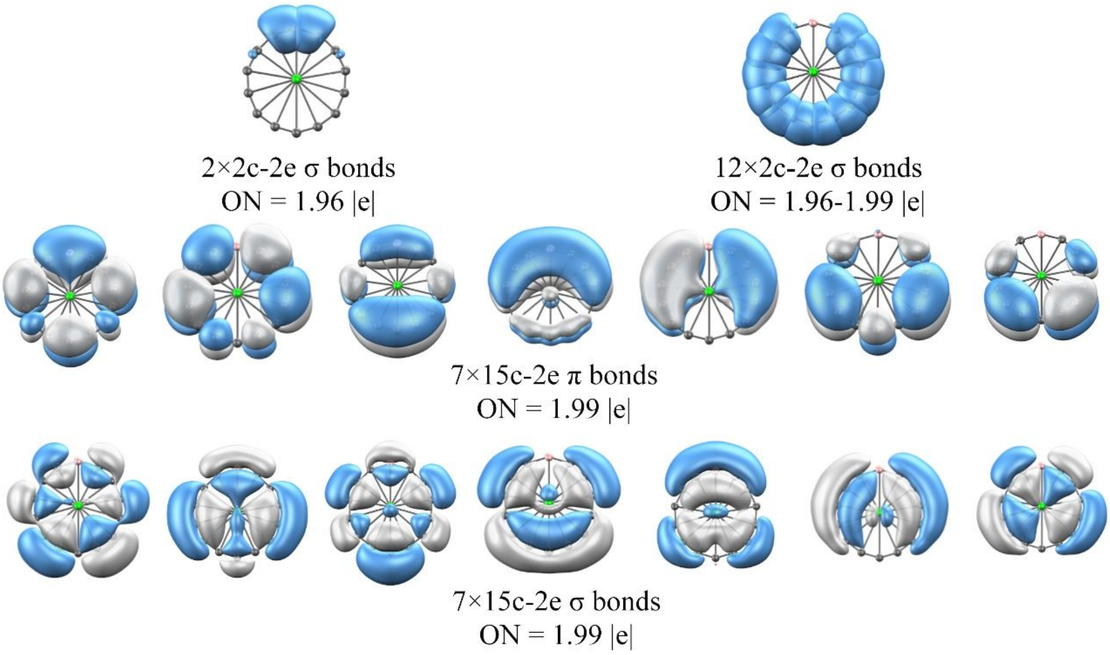

Figure S14. AdNDP orbitals of Sr©BC_{13}^{+} at the M06-2X/def2-TZVP level. ON denotes the occupation number.

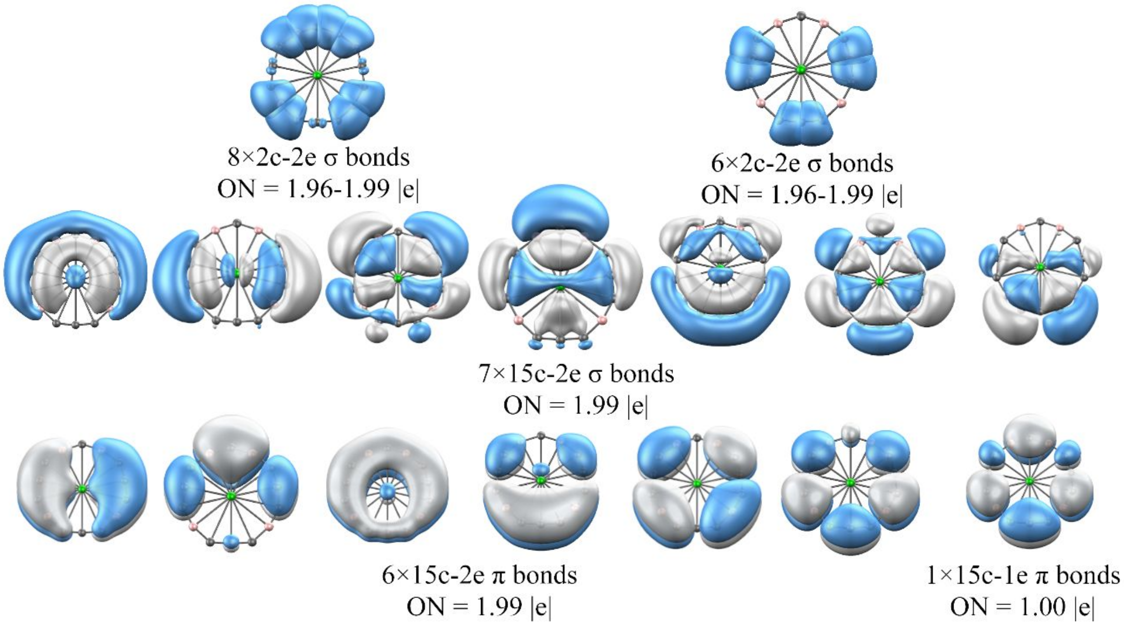

Figure S15. AdNDP orbitals of Ba©$B_4C_{10}^-$ at the M06-2X/def2-TZVP level. ON denotes the occupation number.

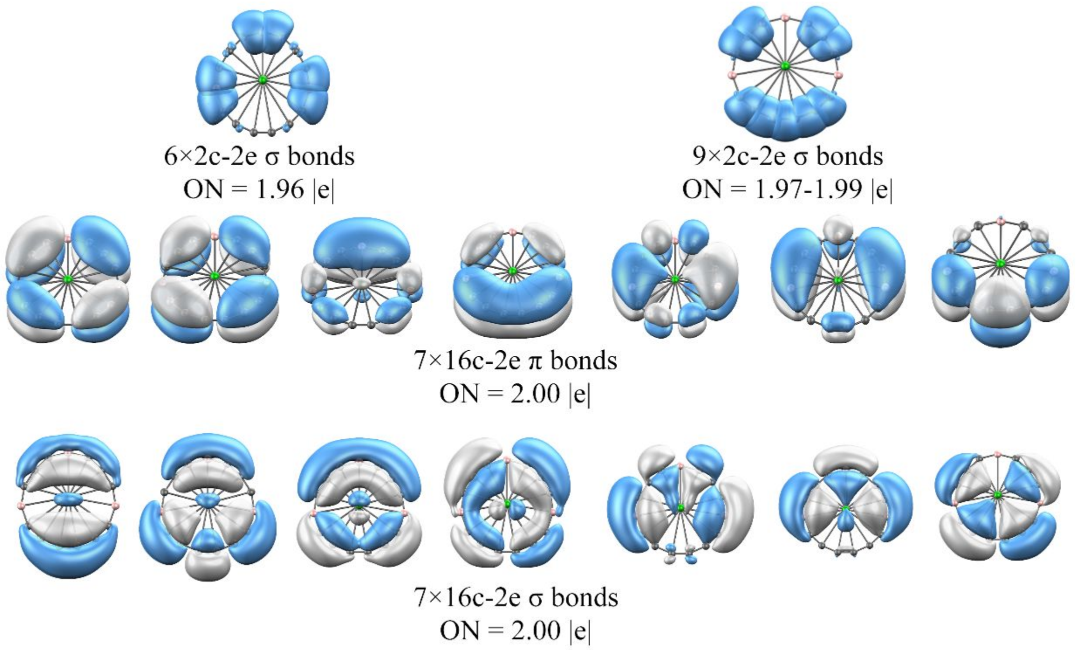

Figure S16. AdNDP orbitals of Ba©$B_3C_{12}^+$ at the M06-2X/def2-TZVP level. ON denotes the occupation number.

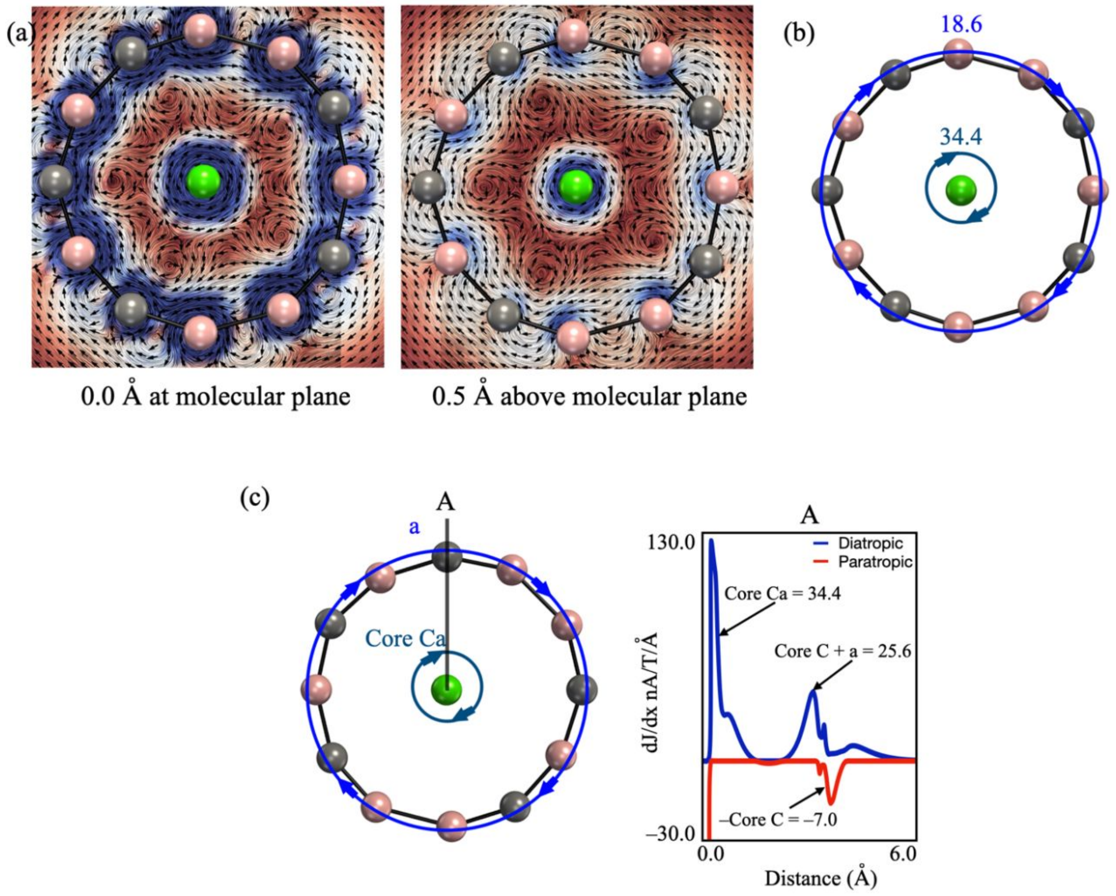

Figure S17. Analysis of ring currents for Ca©$B_7C_5^-$. (a) Plot of vectors at 0.0 and 0.5 Å on the molecular plane. (b) Schematic representation of the ring currents and their strengths in nA/T. (c) The integration plane used in the analysis and the integration profile resulting along this plane, the numbers in each band (integrated values of the flux in nA/T) identify the different contributions.

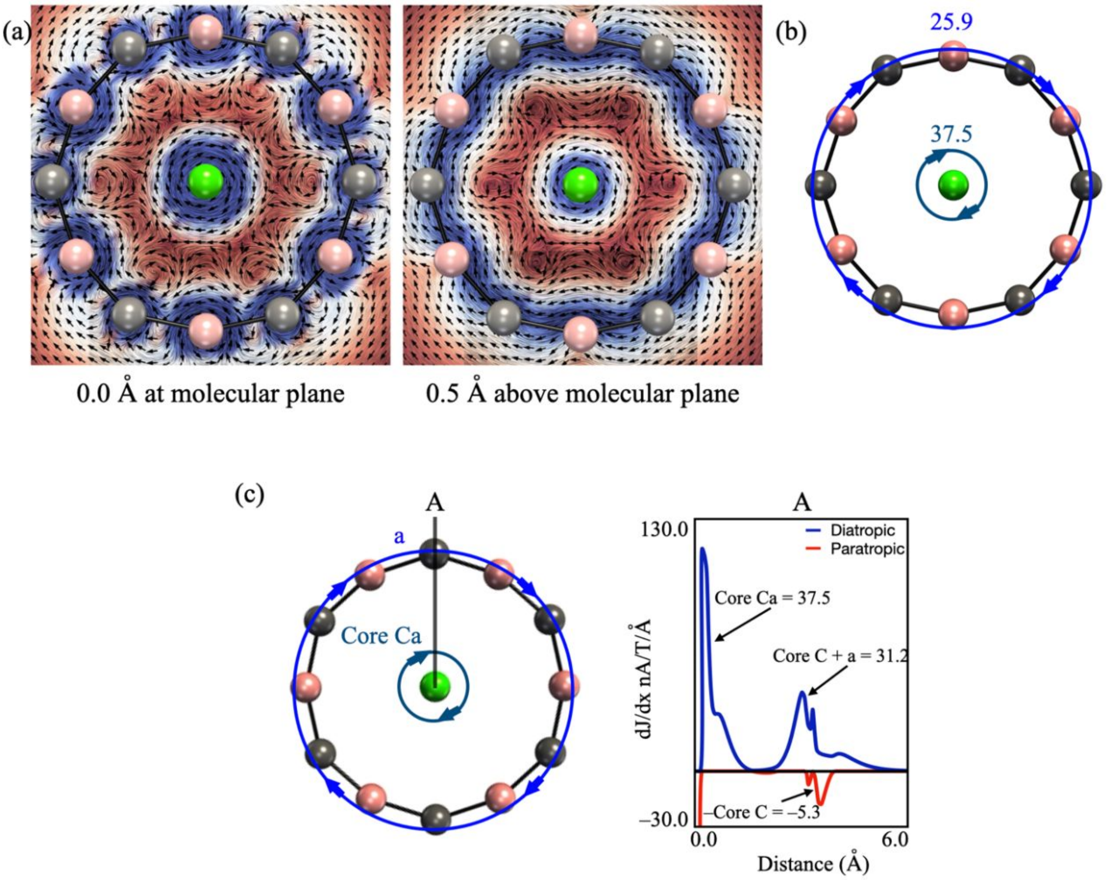

Figure S18. Analysis of ring currents for Ca©$B_6C_6^-$. The details of the figure are similar to those of Figure S17.

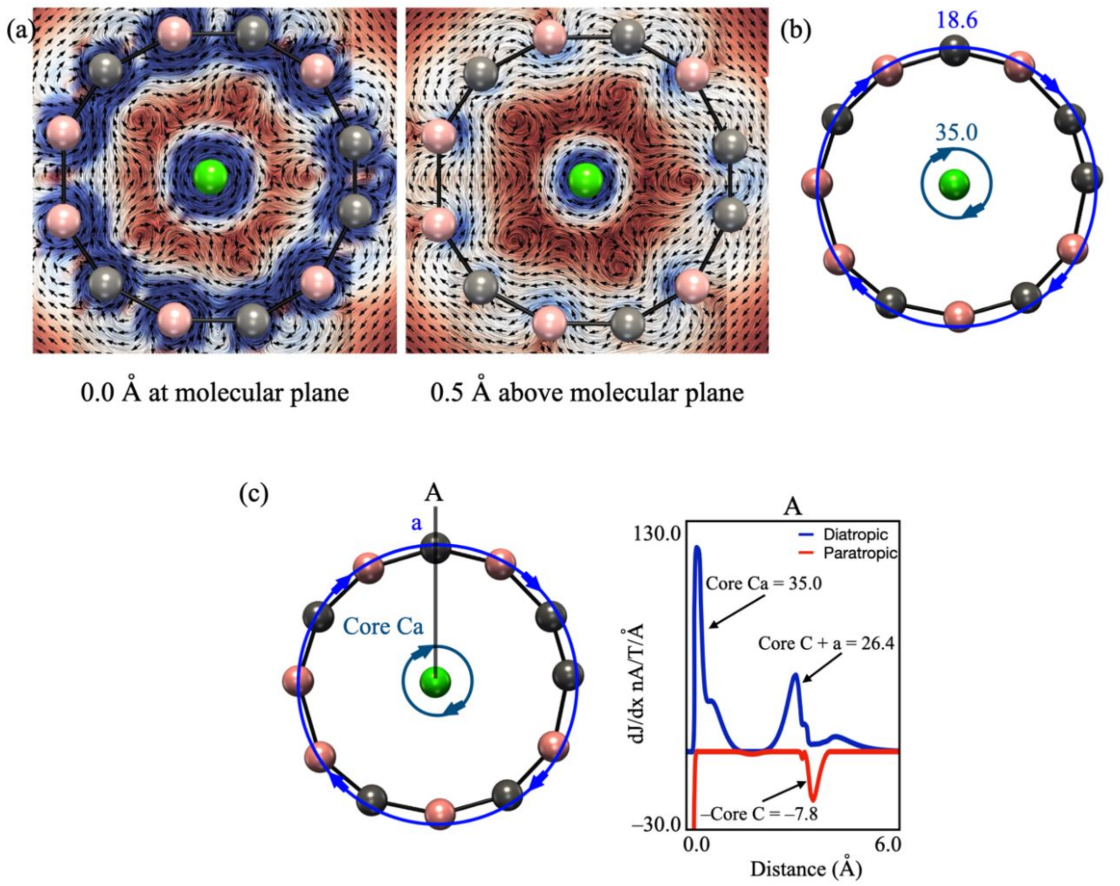

Figure S19. Analysis of ring currents for Ca©B_6C_6. The details of the figure are similar to those of Figure S17.

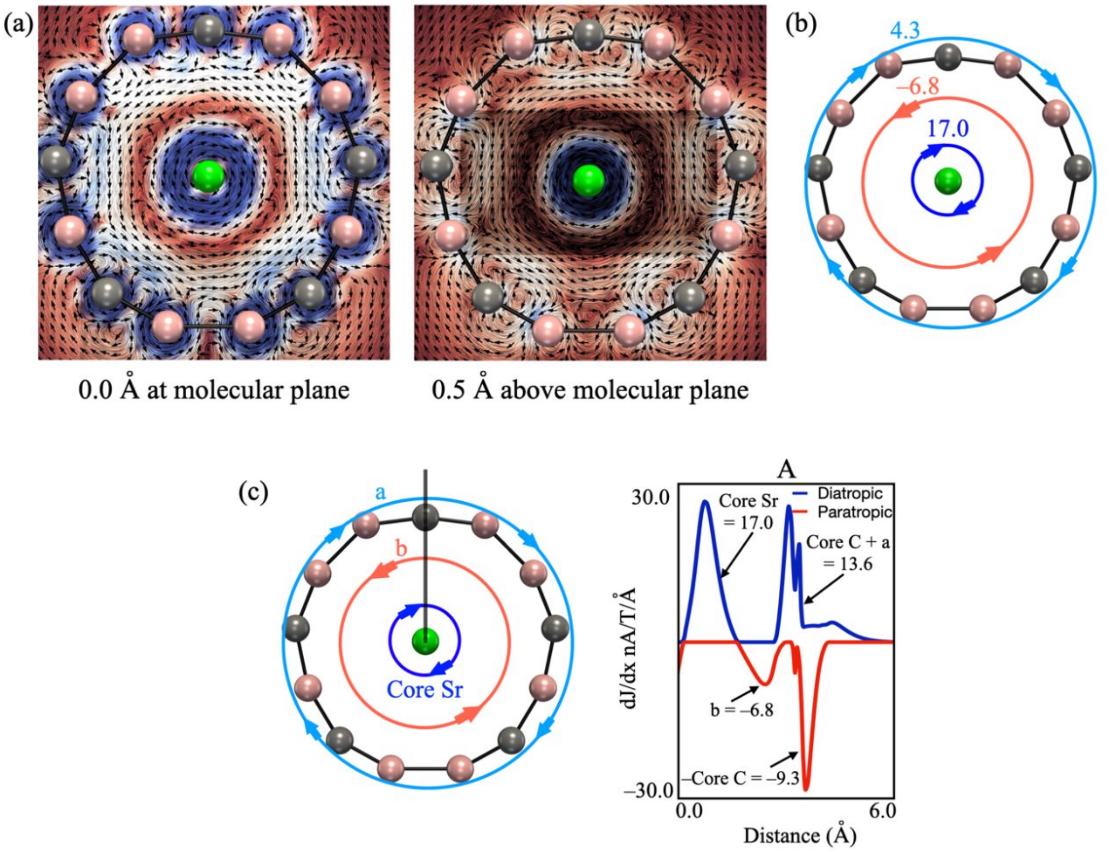

Figure S20. Analysis of ring currents for Sr©$B_8C_5^-$. The details of the figure are similar to those of Figure S17.

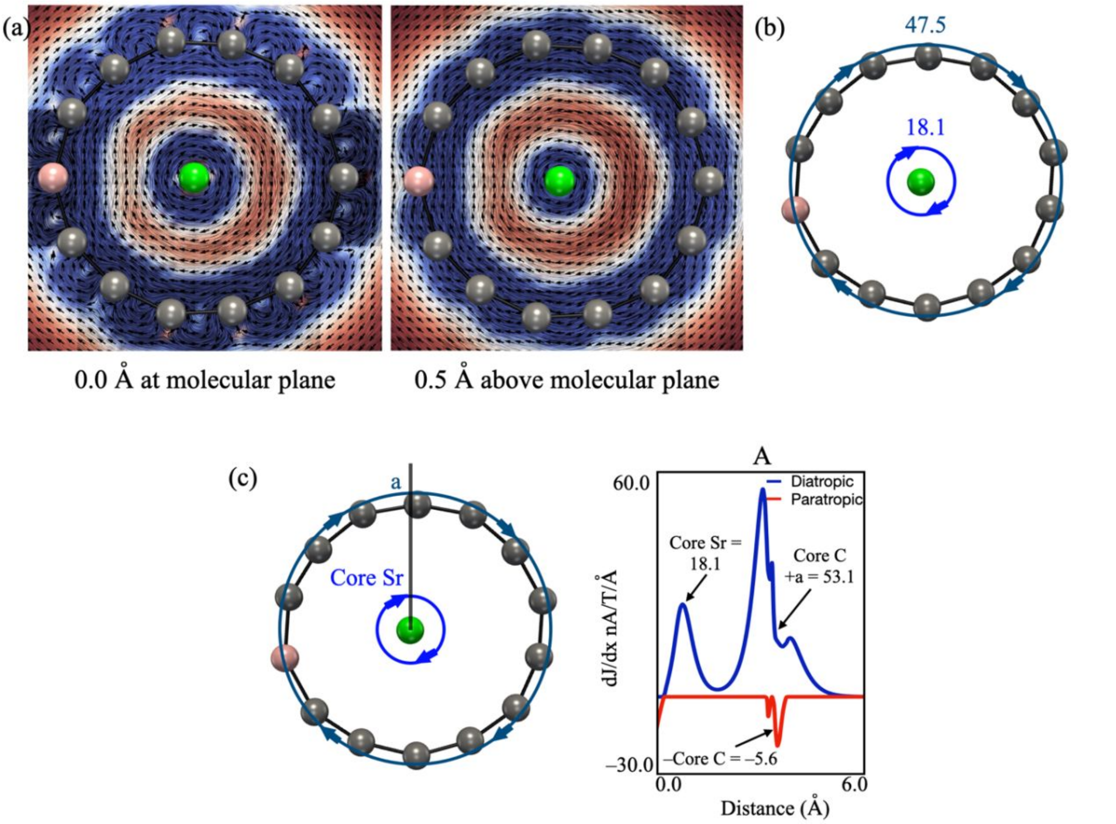

Figure S21. Analysis of ring currents for Sr©BC_{13}^{+}. The details of the figure are similar to those of Figure S17.

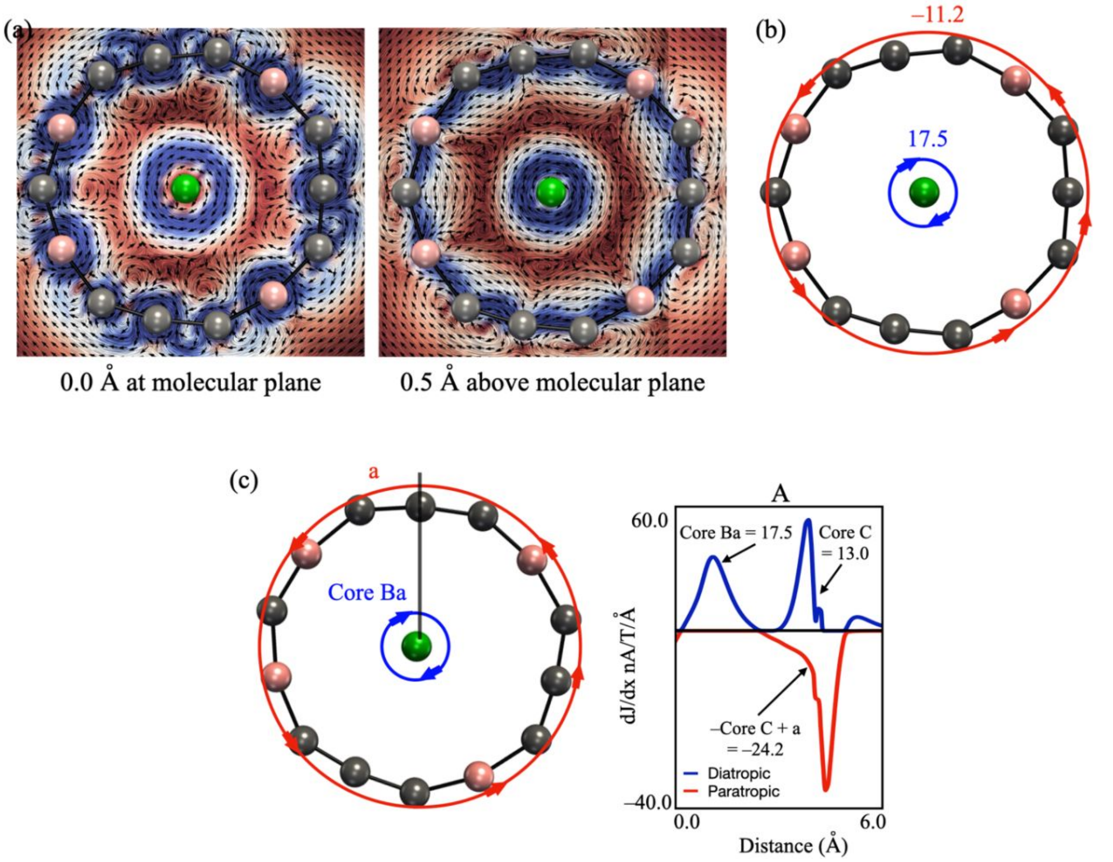

Figure S22. Analysis of ring currents for Ba©$B_4C_{10}^-$. The details of the figure are similar to those of Figure S17.

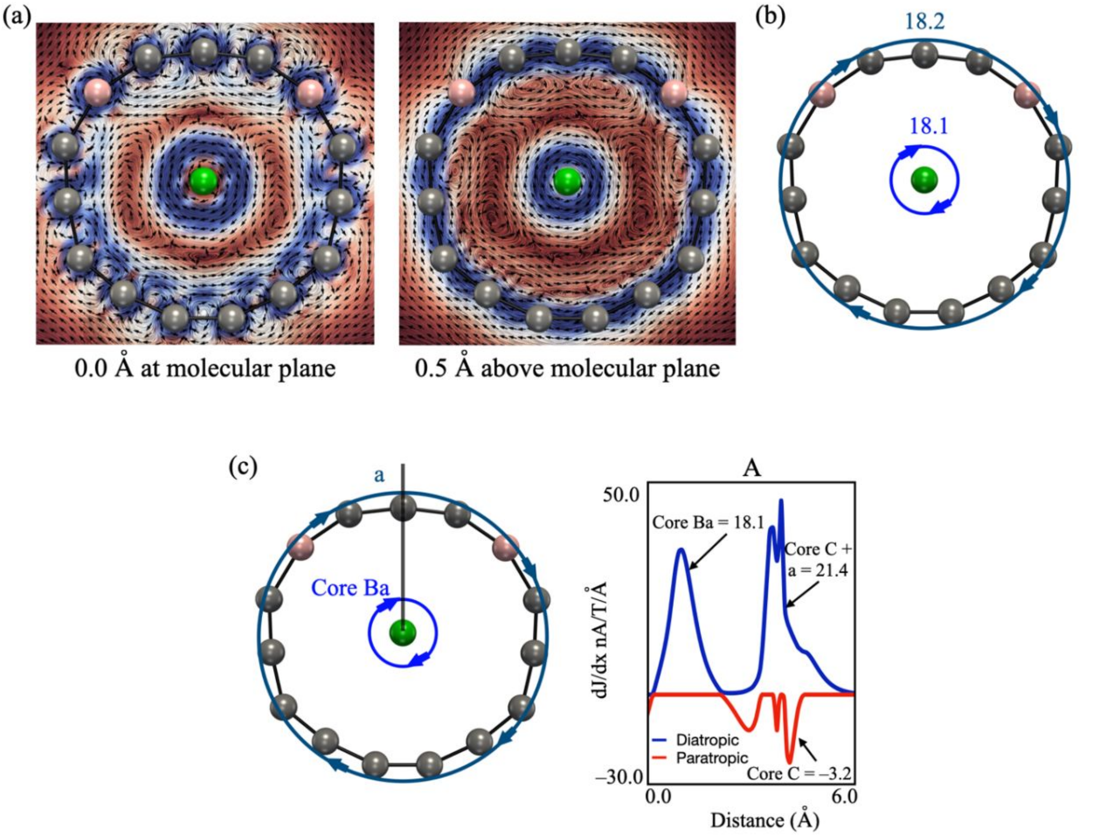

Figure S23. Analysis of ring currents for Ba©$B_2C_{13}^+$. The details of the figure are similar to those of Figure S17.

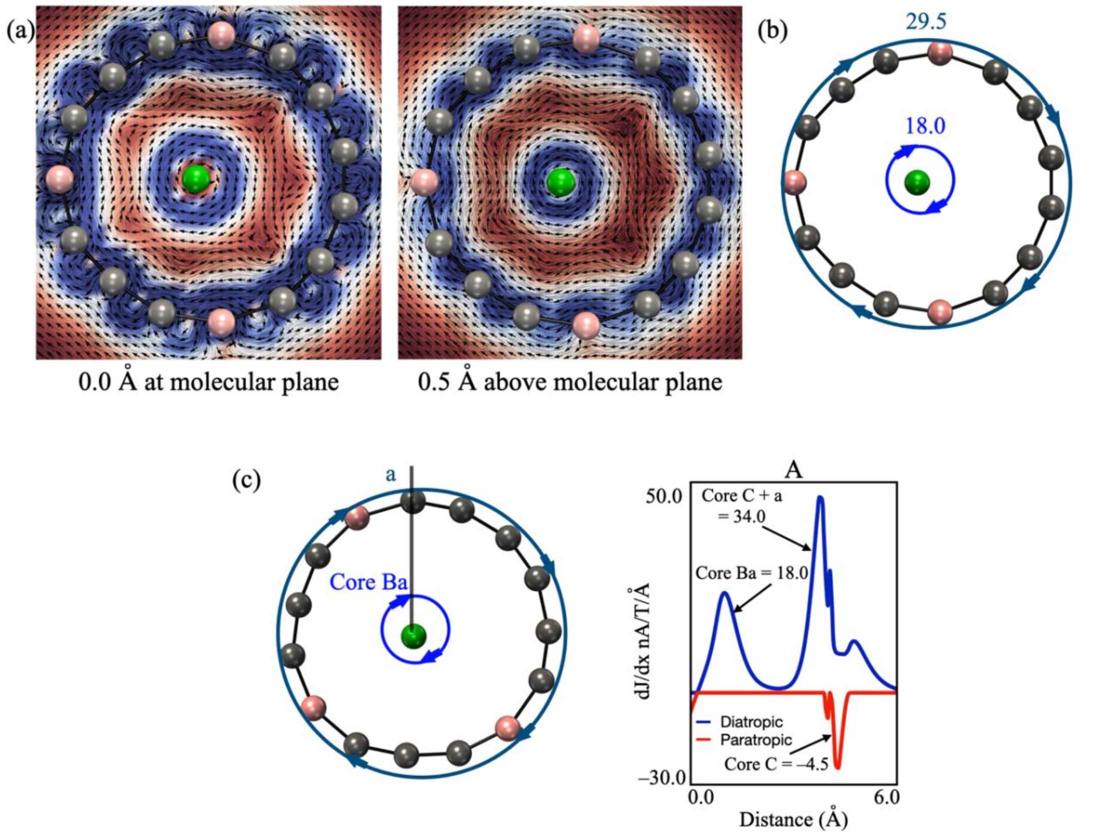

Figure S24. Analysis of ring currents for Ba©$B_3C_{12}^+$. The details of the figure are similar to those of Figure S17.

Table S6. EDA results for the Ca©$B_8C_4^-$ cluster considering Ca and B_8C_4 as interacting fragments in various charge and electronic states at the M06-2X/TZ2P-ZORA level. Energy values are given in kcal/mol.

Energy	Ca^{2+} (S, $3p^6 4s^0$) + $B_8C_4^{3-}$ (D)	Ca^+ (D, $3d_{x^2-y^2}^1$) +$B_8C_4^{2-}$ (T)	Ca^+ (D, $4s^1$) + $B_8C_4^{2-}$ (T)
ΔE_{int}	-713.9	-404.8	-422.0
ΔE_{Pauli}	67.9	84.0	117.0
ΔE_{elstat}	-620.9	-266.6	-297.5
ΔE_{orb}	-160.9	-222.2	-241.5
Energy	Ca^+ (D, $3d_{xy}^1$) + $B_8C_4^{2-}$ (T)	Ca^+ (D, $3d_{z^2}^1$) + $B_8C_4^{2-}$ (T)	Ca (S, $4s^2$) + $B_8C_4^-$ (D)
ΔE_{int}	-358.9	-446.1	-350.4
ΔE_{Pauli}	83.0	165.5	254.7
ΔE_{elstat}	-268.8	-314.1	-195.2
ΔE_{orb}	-173.2	-297.5	-409.9
Energy	Ca (S, $3d_{xy}^2$) + $B_8C_4^-$ (D)		
ΔE_{int}	-320.9		
ΔE_{Pauli}	148.3		
ΔE_{elstat}	-133.1		
ΔE_{orb}	-336.1		

Table S7. EDA results for the Sr©$B_8C_5^-$ cluster considering Sr and B_8C_5 as interacting fragments in various charge and electronic states at the M06-2X/TZ2P-ZORA level. Energy values are given in kcal/mol.

Energy	Sr^{2+} (S, $4p^65s^0$) + $B_8C_5^{3-}$ (D)	Sr^+ (D, $4d_{x^2-y^2}{}^1$) +$B_8C_5^{2-}$ (T)	Sr^+ (D, $5s^1$) + $B_8C_5^{2-}$ (T)
ΔE_{int}	-655.0	-378.8	-437.6
ΔE_{Pauli}	62.4	73.5	105.5
ΔE_{elstat}	-528.6	-251.4	-275.7
ΔE_{orb}	-126.4	-201.0	-267.4
Energy	Sr^+ (D, $4d_{xy}{}^1$) + $B_8C_5^{2-}$ (T)	Sr^+ (D, $4d_{z^2}{}^1$) + $B_8C_5^{2-}$ (T)	Sr (S, $5s^2$) + $B_8C_5^-$ (D)
ΔE_{int}	-369.1	-461.5	-218.0
ΔE_{Pauli}	73.0	61.1	293.7
ΔE_{elstat}	-249.5	-236.3	-195.4
ΔE_{orb}	-192.6	-286.3	-316.3
Energy	Sr (S, $4d_{xy}{}^2$) + $B_8C_5^-$ (D)		
ΔE_{int}	-385.3		
ΔE_{Pauli}	113.0		
ΔE_{elstat}	-101.6		
ΔE_{orb}	-396.7		

Table S8. EDA results for the Sr©BC_{13}^+ cluster considering Sr and BC_{13} as interacting fragments in various charge and electronic states at the M06-2X/TZ2P-ZORA level. Energy values are given in kcal/mol.

Energy	Sr^{2+} (S, $4p^65s^0$) + BC_{13}^- (S)	Sr^+ (D, $4d_{x^2-y^2}{}^1$) + BC_{13} (D)	Sr^+ (D, $4d_{xy}{}^1$) + BC_{13} (D)
ΔE_{int}	-274.6	-151.3	-242.0
ΔE_{Pauli}	40.3	93.5	62.3
ΔE_{elstat}	-207.9	-52.0	-51.2
ΔE_{orb}	-107.1	-192.8	-253.1
Energy	Sr^+ (D, $5s^1$) + BC_{13} (D)	Sr (T, $4d_{xy}{}^1 4d_{x^2-y^2}{}^1$) + BC_{13}^+ (T)	
ΔE_{int}	-374.2	-481.1	
ΔE_{Pauli}	114.0	117.3	
ΔE_{elstat}	-83.3	-106.6	
ΔE_{orb}	-404.9	-491.8	

Table S9. EDA results for the Ba©$B_4C_{10}^-$ cluster considering Ba and B_4C_{10} as interacting fragments in various charge and electronic states at the M06-2X/TZ2P-ZORA level. Energy values are given in kcal/mol.

Energy	Ba^{2+} (S, $5p^6 6s^0$) + $B_4C_{10}^{3-}$ (D)	Ba^+ (D, $5d_{xy}^1$) + $B_4C_{10}^{2-}$ (T)	Ba^+ (D, $5d_{x^2-y^2}^1$) + $B_4C_{10}^{2-}$ (T)
ΔE_{int}	-615.7	-359.6	-366.0
ΔE_{Pauli}	70.9	139.1	150.2
ΔE_{elstat}	-569.3	-282.0	-280.9
ΔE_{orb}	-117.2	-216.6	-235.4
Energy	Ba^+ (D, $6s^1$) + $B_4C_{10}^{2-}$ (T)	Ba (T, $5d_{xy}^1 5d_{x^2-y^2}^1$) + $B_4C_{10}^-$ (D)	
ΔE_{int}	-408.2	-333.7	
ΔE_{Pauli}	171.7	311.5	
ΔE_{elstat}	-295.6	-246.8	
ΔE_{orb}	-284.3	-398.3	

Table S10. EDA results for the Ba©$B_3C_{12}^+$ cluster considering Ba and B_3C_{12} as interacting fragments in various charge and electronic states at the M06-2X/TZ2P-ZORA level. Energy values are given in kcal/mol.

Energy	Ba^{2+} (S, $5p^6 6s^0$) + $B_3C_{12}^-$ (S)	Ba^+ (D, $5d_{xy}{}^1$) + B_3C_{12} (D)	Ba^+ (D, $5d_{x^2-y^2}{}^1$) + B_3C_{12} (D)
ΔE_{int}	-260.7	-231.4	-155.7
ΔE_{Pauli}	39.6	101.8	107.9
ΔE_{elstat}	-205.8	-80.9	-78.9
ΔE_{orb}	-94.5	-252.2	-184.8
Energy	Ba^+ (D, $6s^1$) + B_3C_{12} (D)	Ba (T, $5d_{xy}{}^1 5d_{x^2-y^2}{}^1$) + $B_3C_{12}^+$ (T)	
ΔE_{int}	-417.8	-358.9	
ΔE_{Pauli}	106.3	256.7	
ΔE_{elstat}	-63.2	-206.6	
ΔE_{orb}	-460.9	-409.0	

Table S11. EDA results for the Sr©$B_8C_5^-$ cluster considering Sr and B_8C_5 with Sr^{2+} (S, $4p^65s^0$) + $B_8C_5^{3-}$ (D) as interacting fragments at the M06-2X/TZ2P-ZORA level. Energy values are given in kcal/mol.

Energy	Interaction	Sr^{2+} (S, $3p^64s^0$) + $C_5B_8^{3-}$ (D)
ΔE_{int}		-655.0
$\Delta E_{\mathrm{Pauli}}$		62.4
$\Delta E_{\mathrm{elstat}}$[a]		-528.6 (80.7%)
ΔE_{orb}[a]		-126.4 (19.3%)
$\Delta E_{\mathrm{orb(1)}}$[b]	$B_8C_5^{3-} \rightarrow Sr^{2+}$ ($4d_\sigma$) σ backdonation	-28.2 (22.3%)
$\Delta E_{\mathrm{orb(2)}}$[b]	$B_8C_5^{3-} \rightarrow Sr^{2+}$ ($4d_{\sigma'}$) σ backdonation	-27.8 (21.9%)
$\Delta E_{\mathrm{orb(3)}}$[b]	$B_8C_5^{3-} \rightarrow Sr^{2+}$ (5s) σ backdonation	-16.9 (13.3%)
$\Delta E_{\mathrm{orb(4)}}$[b]	$B_8C_5^{3-} \rightarrow Sr^{2+}$ ($5p_\sigma$) σ backdonation	-8.6 (6.8%)
$\Delta E_{\mathrm{orb(5)}}$[b]	$B_8C_5^{3-} \rightarrow Sr^{2+}$ ($5p_{\sigma'}$) σ backdonation	-8.4 (6.7%)
$\Delta E_{\mathrm{orb(6)}}$[b]	$B_8C_5^{3-} \rightarrow Sr^{2+}$ ($4d_\pi$) π backdonation	-7.3 (5.8%)
$\Delta E_{\mathrm{orb(7)}}$[b]	$B_8C_5^{3-} \rightarrow Sr^{2+}$ ($4d_{\pi'}$) π backdonation	-6.9 (5.5%)
$\Delta E_{\mathrm{orb(rest)}}$[b]		-22.3 (17.6%)

[a]The percentage contribution with respect to total attraction is given in parentheses; [b]The percentage contribution in parentheses is given with respect to total orbital interaction.

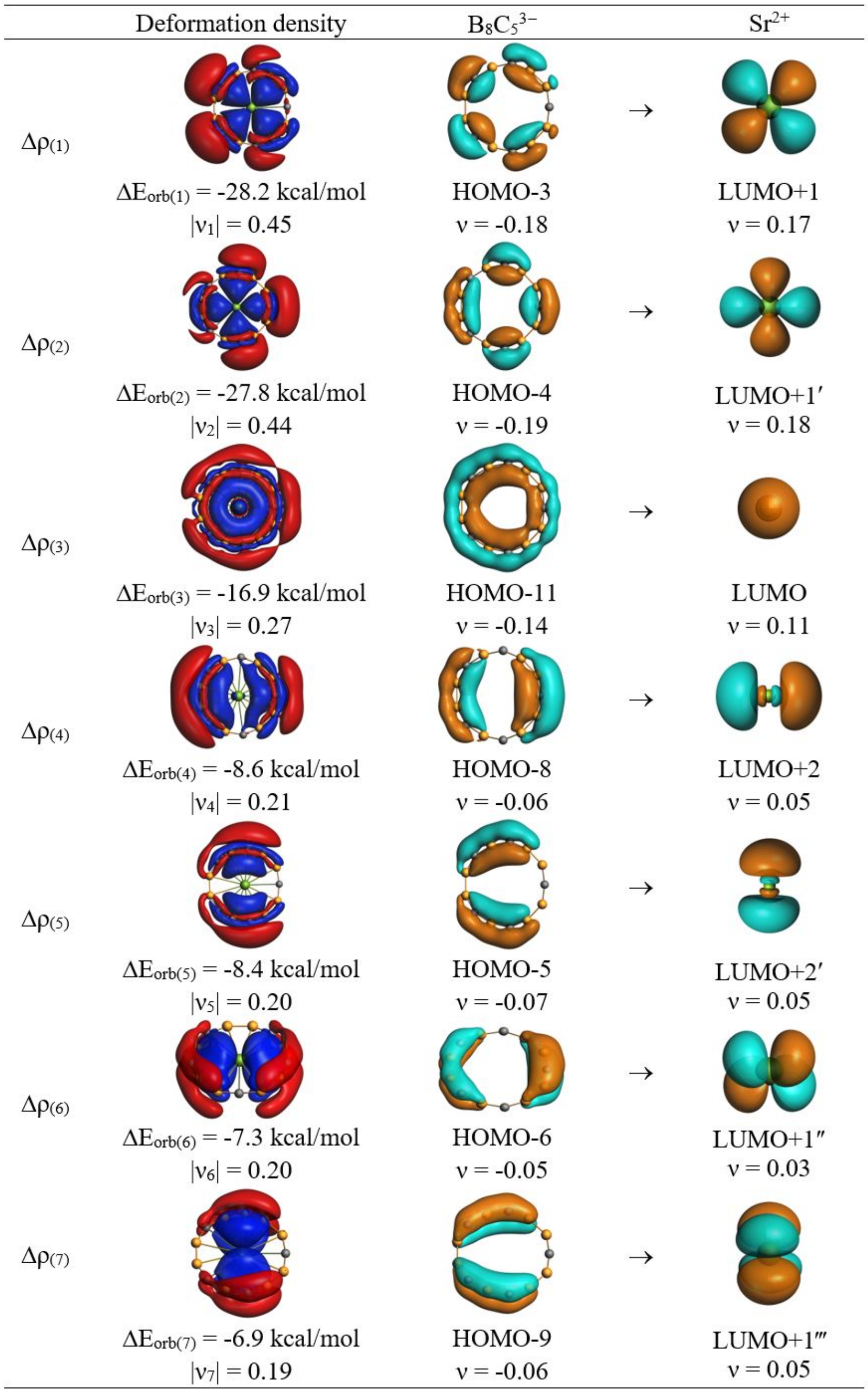

Figure S25. Plot of deformation densities, $\Delta\rho_{(1)-(7),}$ representing the sum of α and β electronic charge related to $\Delta E_{orb(1)-(7)}$ and the interacting orbitals of fragments in the Sr©$B_8C_5^-$ cluster at the M06-2X/TZ2P-ZORA level, with Sr^{2+} (S, $4p^65s^0$) + $B_8C_5^{3-}$ (D) as interacting fragments. Charge flow direction in the deformation densities is indicated from red to blue. The isovalue for $\Delta\rho_{(1)-(7)}$ is 0.0001 au.

Table S12. EDA results for the Sr©BC_{13}^{+} cluster considering Sr and BC_{13} with Sr^{2+} (S, $4p^65s^0$) + BC_{13}^{-} (S) as interacting fragments at the M06-2X/TZ2P-ZORA level. Energy values are given in kcal/mol.

Energy	Interaction	Sr^{2+} (S, $4p^65s^0$) + BC_{13}^{-} (S)
ΔE_{int}		-274.6
ΔE_{Pauli}		40.3
ΔE_{elstat}[a]		-207.9 (66.0%)
ΔE_{orb}[a]		-107.1 (34.0%)
$\Delta E_{orb(1)}$[b]	$BC_{13}^{-} \rightarrow Sr^{2+}$ ($4d_{\sigma}$) σ donation	-22.1 (20.6%)
$\Delta E_{orb(2)}$[b]	$BC_{13}^{-} \rightarrow Sr^{2+}$ ($4d_{\sigma'}$) σ donation	-19.8 (18.5%)
$\Delta E_{orb(3)}$[b]	$BC_{13}^{-} \rightarrow Sr^{2+}$ (5s) σ donation	-13.3 (12.4%)
$\Delta E_{orb(4)}$[b]	$BC_{13}^{-} \rightarrow Sr^{2+}$ ($4f_{\sigma}$) σ donation	-9.0 (8.4%)
$\Delta E_{orb(5)}$[b]	$BC_{13}^{-} \rightarrow Sr^{2+}$ ($4f_{\sigma'}$) σ donation	-6.8 (6.3%)
$\Delta E_{orb(6)}$[b]	$BC_{13}^{-} \rightarrow Sr^{2+}$ ($5p_{\sigma}$) σ donation	-6.3 (5.9%)
$\Delta E_{orb(7)}$[b]	$BC_{13}^{-} \rightarrow Sr^{2+}$ ($5p_{\sigma'}$) σ donation	-6.2 (5.8%)
$\Delta E_{orb(rest)}$[b]		-23.6 (22.0%)

[a]The percentage contribution with respect to total attraction is given in parentheses; [b]The percentage contribution in parentheses is given with respect to total orbital interaction.

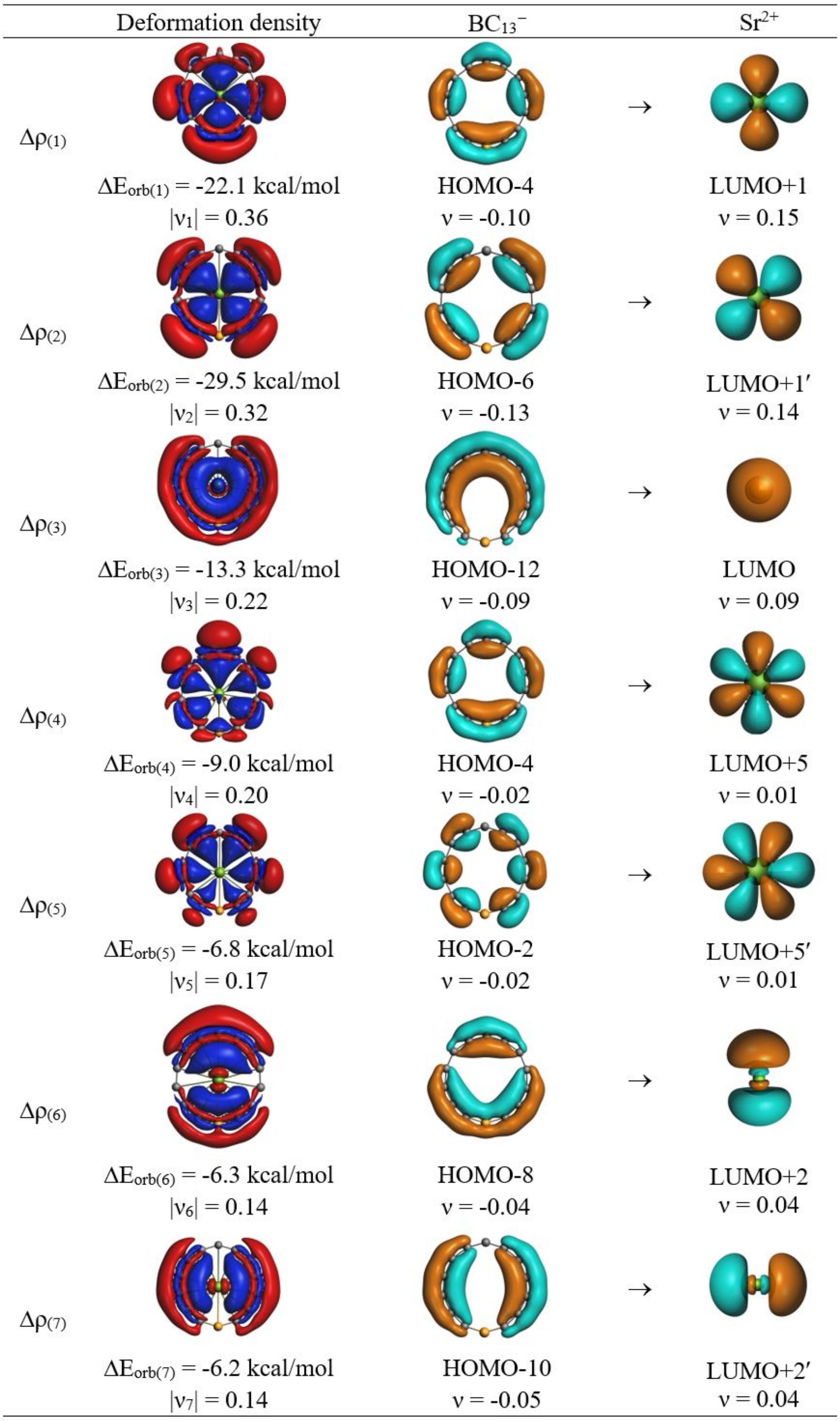

Figure S26. Plot of deformation densities, $\Delta\rho_{(1)\text{-}(7)}$, representing the sum of α and β electronic charge related to $\Delta E_{orb(1)\text{-}(7)}$ and the interacting orbitals of fragments in the Sr©BC_{13}^{+} cluster at the M06-2X/TZ2P-ZORA level, with Sr^{2+} (S, $4p^65s^0$) + BC_{13}^{-} (S) as interacting fragments. Charge flow direction in the deformation densities is indicated from red to blue. The isovalue for $\Delta\rho_{(1)\text{-}(7)}$ is 0.0001 au.

Table S13. EDA results for the Ba©$B_4C_{10}^-$ cluster considering Ba and B_4C_{10} with Ba^{2+} (S, $5p^66s^0$) + $B_4C_{10}^{3-}$ (D) as interacting fragments at the M06-2X/TZ2P-ZORA level. Energy values are given in kcal/mol.

Energy	Interaction	Ba^{2+} (S, $5p^66s^0$) + $B_4C_{10}^{3-}$ (D)
ΔE_{int}		-615.7
ΔE_{Pauli}		70.9
ΔE_{elstat}[a]		-569.3 (82.9%)
ΔE_{orb}[a]		-117.2 (17.1%)
$\Delta E_{orb(1)}$[b]	$B_4C_{10}^{3-} \rightarrow Ba^{2+}$ ($5d_\sigma$) σ donation	-27.1 (23.1%)
$\Delta E_{orb(2)}$[b]	$B_4C_{10}^{3-} \rightarrow Ba^{2+}$ ($5d_{\sigma'}$) σ donation	-23.2 (19.8%)
$\Delta E_{orb(3)}$[b]	$B_4C_{10}^{3-} \rightarrow Ba^{2+}$ (6s) σ donation	-13.6 (11.6%)
$\Delta E_{orb(4)}$[b]	$B_4C_{10}^{3-} \rightarrow Ba^{2+}$ ($4f_\sigma$) σ donation	-12.2 (10.4%)
$\Delta E_{orb(5)}$[b]	$B_4C_{10}^{3-} \rightarrow Ba^{2+}$ ($4f_{\sigma'}$) σ donation	-11.4 (9.7%)
$\Delta E_{orb(6)}$[b]	$B_4C_{10}^{3-} \rightarrow Ba^{2+}$ ($5d_\pi$) π donation	-6.6 (5.6%)
$\Delta E_{orb(7)}$[b]	$B_4C_{10}^{3-} \rightarrow Ba^{2+}$ ($5d_{\pi'}$) π donation	-5.5 (4.7%)
$\Delta E_{orb(rest)}$[b]		-17.6 (15.0%)

[a]The percentage contribution with respect to total attraction is given in parentheses; [b]The percentage contribution in parentheses is given with respect to total orbital interaction.

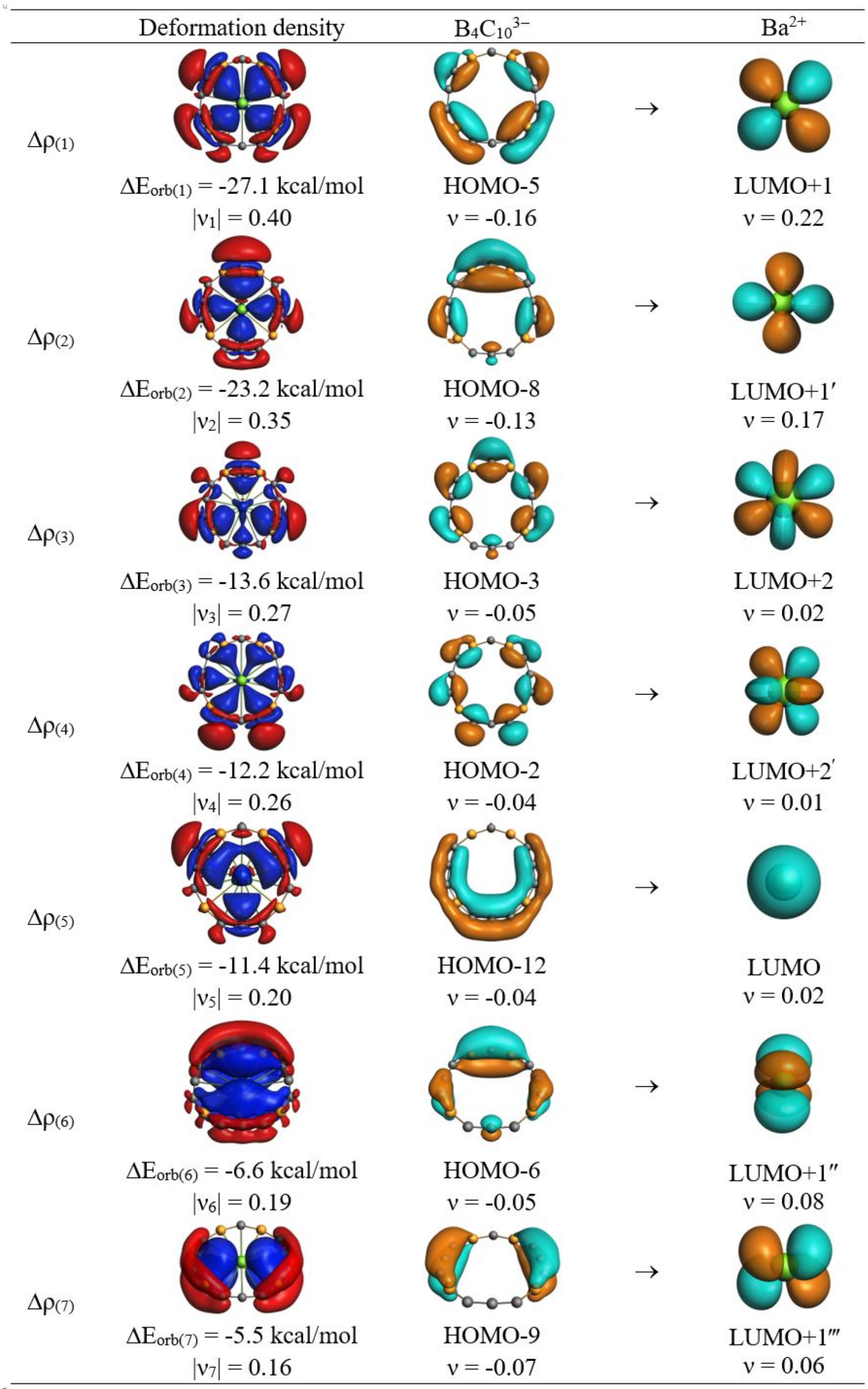

Figure S27. Plot of deformation densities, $\Delta\rho_{(1)\text{-}(7),}$ representing the sum of α and β electronic charge related to $\Delta E_{\text{orb}(1)\text{-}(7)}$ and the interacting orbitals of fragments in the Ba©$B_4C_{10}^-$ cluster at the M06-2X/TZ2P-ZORA level, with Ba^{2+} (S, $5p^66s^0$) + $B_4C_{10}^{3-}$ (D) as interacting fragments. Charge flow direction in the deformation densities is indicated from red to blue. The isovalue for $\Delta\rho_{(1)\text{-}(7)}$ is 0.0001 au.

Table S14. EDA results for the Ba©$B_3C_{12}^+$ cluster considering Ba and B_4C_{10} with Ba and B_3C_{12} with Ba^{2+} (S, $5p^6 6s^0$) + $B_3C_{12}^-$ (S) as interacting fragments at the M06-2X/TZ2P-ZORA level. Energy values are given in kcal/mol.

Energy	Interaction	Ba^{2+} (S, $5p^6 6s^0$) + $B_3C_{12}^-$ (S)
ΔE_{int}		-260.7
ΔE_{Pauli}		39.6
ΔE_{elstat}[a]		-205.8 (68.5%)
ΔE_{orb}[a]		-94.5 (31.5%)
$\Delta E_{orb(1)}$[b]	$B_3C_{12}^- \rightarrow Ba^{2+}$ ($5d_\sigma$) σ donation	-22.7 (24.0%)
$\Delta E_{orb(2)}$[b]	$B_3C_{12}^- \rightarrow Ba^{2+}$ ($5d_{\sigma'}$) σ donation	-17.4 (18.4%)
$\Delta E_{orb(3)}$[b]	$B_3C_{12}^- \rightarrow Ba^{2+}$ (6s) σ donation	-10.8 (11.4%)
$\Delta E_{orb(4)}$[b]	$B_3C_{12}^- \rightarrow Ba^{2+}$ ($4f_\sigma$) σ donation	-6.7 (7.1%)
$\Delta E_{orb(5)}$[b]	$B_3C_{12}^- \rightarrow Ba^{2+}$ ($4f_{\sigma'}$) σ donation	-6.3 (6.6%)
$\Delta E_{orb(6)}$[b]	$B_3C_{12}^- \rightarrow Ba^{2+}$ ($5d_\pi$) π donation	-4.7 (5.0%)
$\Delta E_{orb(7)}$[b]	$B_3C_{12}^- \rightarrow Ba^{2+}$ ($5d_{\pi'}$) π donation	-4.6 (4.9%)
$\Delta E_{orb(rest)}$[b]		-21.4 (22.6%)

[a]The percentage contribution with respect to total attraction is given in parentheses; [b]The percentage contribution in parentheses is given with respect to total orbital interaction.

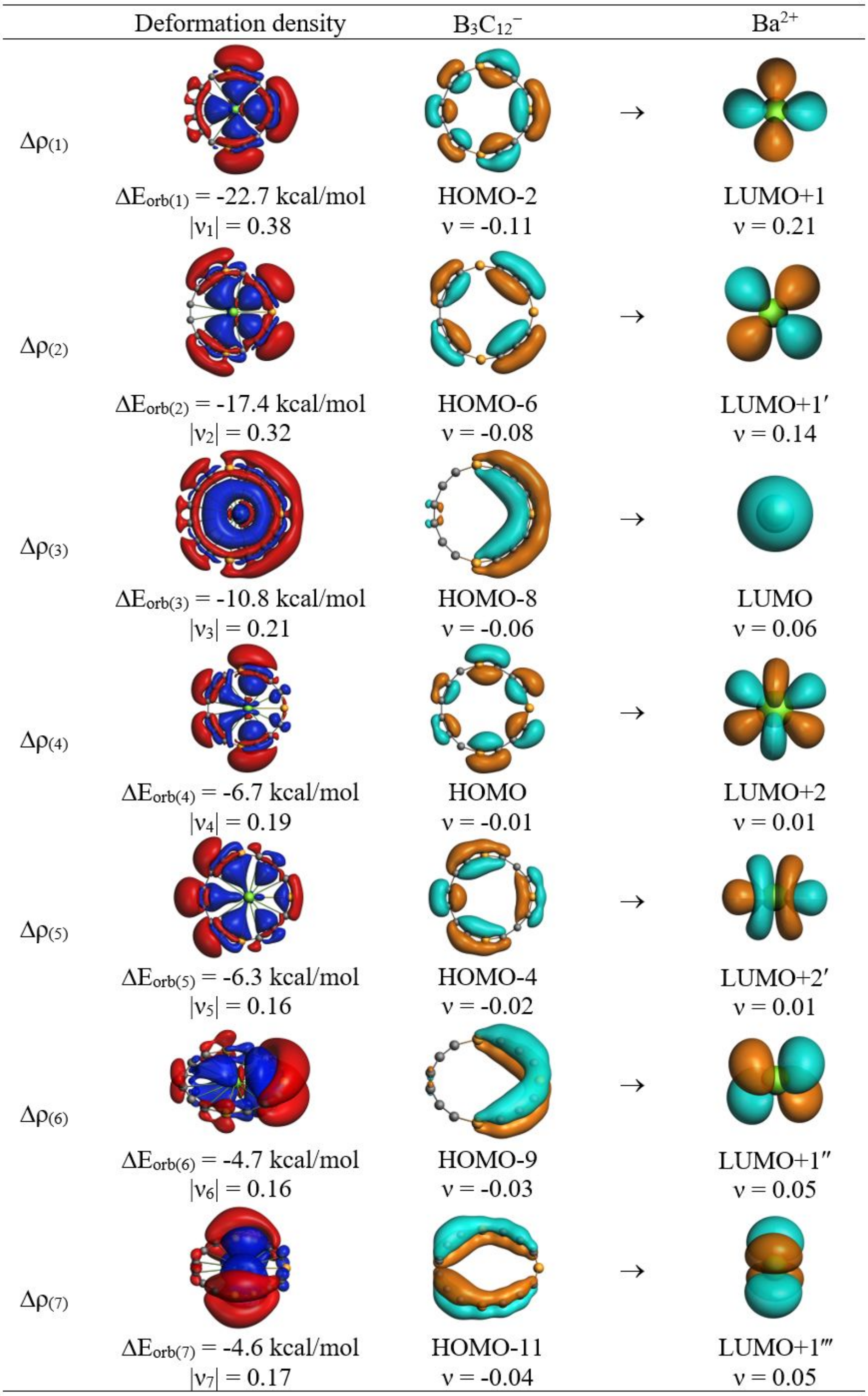

Figure S28. Plot of deformation densities, $\Delta\rho_{(1)-(7),}$ representing the sum of α and β electronic charge related to $\Delta E_{orb(1)-(7)}$ and the interacting orbitals of fragments in the Ba©$B_3C_{12}^+$ cluster at the M06-2X/TZ2P-ZORA level, with Ba^{2+} (S, $5p^66s^0$) + $B_3C_{12}^-$ (D) as interacting fragments. Charge flow direction in the deformation densities is indicated from red to blue. The isovalue for $\Delta\rho_{(1)-(7)}$ is 0.0001 au.

Table S15. The EDA results of the Na©C_{14}^+ cluster considering Na^+ (S, $3s^03p^0$) + C_{14} (S) as interacting fragments at the M06-2X/TZ2P-ZORA level. Energy values are given in kcal/mol.

Energy	Interaction	Na^+ (S, $3s^03p^0$) + C_{14} (S)
ΔE_{int}		-22.2
ΔE_{Pauli}		3.6
ΔE_{elstat}[a]		-1.3 (5.0%)
ΔE_{orb}[a]		-24.5 (95.0%)
$\Delta E_{orb(1)}$[b]	$C_{14} \rightarrow Na^+$ (3s) donation	-4.3 (17.6%)
$\Delta E_{orb(2)}$[b]	$C_{14} \rightarrow Na^+$ (3p) donation	-2.8 (11.4%)
$\Delta E_{orb(3)}$[b]	$C_{14} \rightarrow Na^+$ (3p) donation	-2.8 (11.4%)
$\Delta E_{orb(4)}$[b]	$C_{14} \rightarrow Na^+$ (3d) donation	-2.0 (8.2%)
$\Delta E_{orb(5)}$[b]	$C_{14} \rightarrow Na^+$ (3d) donation	-2.0 (8.2%)
$\Delta E_{orb(rest)}$[b]		-10.6 (43.3%)

[a]The percentage contribution with respect to total attraction is given in parentheses; [b]The percentage contribution in parentheses is given with respect to total orbital interaction.

Table S16. The EDA results of the Cs©C_{18}^+ cluster considering Cs^+ (S, $6s^06p^0$) + C_{18} (S) as interacting fragments at the M06-2X/TZ2P-ZORA level. Energy values are given in kcal/mol.

Energy	Interaction	Cs^+ (S, $6s^06p^0$) + C_{18} (S)
ΔE_{int}		-14.9
ΔE_{Pauli}		2.1
ΔE_{elstat}[a]		-3.0 (17.6%)
ΔE_{orb}[a]		-14.0 (82.4%)
$\Delta E_{orb(1)}$[b]	$C_{18} \rightarrow Cs^+$ (5d) donation	-2.0 (14.3%)
$\Delta E_{orb(2)}$[b]	$C_{18} \rightarrow Cs^+$ (5d) donation	-2.0 (14.3%)
$\Delta E_{orb(3)}$[b]	$C_{18} \rightarrow Cs^+$ (6s) donation	-2.0 (14.3%)
$\Delta E_{orb(4)}$[b]	$C_{18} \rightarrow Cs^+$ (6p) σ donation	-1.2 (8.6%)
$\Delta E_{orb(5)}$[b]	$C_{18} \rightarrow Cs^+$ (6p) σ donation	-1.2 (8.6%)
$\Delta E_{orb(rest)}$[b]		-5.6 (40.0%)

[a]The percentage contribution with respect to total attraction is given in parentheses; [b]The percentage contribution in parentheses is given with respect to total orbital interaction.

II. Cartesian coordinates of lowest-energy phAe structures at the M06-2X/def2-TZVP level. Total energy (HF) and the lowest vibrational frequencies (ν_1) are given.

Ca©$B_8C_4^-$
HF = -1028.7290579 a.u.
Frequencies ν_1= 97 cm^{-1}

B	1.352352000	2.450339000	0.000000000
B	-1.352352000	-2.450339000	0.000000000
B	-2.450339000	-1.352352000	-0.000000000
B	2.450339000	1.352352000	-0.000000000
B	-2.450339000	1.352352000	0.000000000
B	1.352352000	-2.450339000	-0.000000000
B	2.450339000	-1.352352000	0.000000000
B	-1.352352000	2.450339000	-0.000000000
C	0.000000000	2.748144000	-0.000000000
C	-0.000000000	-2.748144000	-0.000000000
C	-2.748144000	-0.000000000	-0.000000000
C	2.748144000	-0.000000000	-0.000000000
Ca	0.000000000	0.000000000	0.000000000

Ca©$B_7C_5^-$
HF = -1042.0389348 a.u.
Frequencies ν_1= 83 cm^{-1}

B	-0.000000000	0.000000000	-2.688114000
C	0.000000000	1.377904000	-2.394200000
C	-0.000000000	-1.377904000	-2.394200000
B	0.000000000	2.372770000	-1.466702000
B	-0.000000000	-2.372770000	-1.466702000
B	0.000000000	2.794901000	0.067915000
Ca	0.000000000	0.000000000	0.010794000
C	0.000000000	0.000000000	2.745866000
B	-0.000000000	-1.320467000	2.325417000
B	0.000000000	1.320467000	2.325417000
C	-0.000000000	-2.368222000	1.351132000
C	0.000000000	2.368222000	1.351132000
B	-0.000000000	-2.794901000	0.067915000

Ca©$B_6C_6^-$
HF = -1055.2394622 a.u.
Frequencies ν_1= 82 cm^{-1}

B	-0.000000000	2.664270000	0.000000000
C	2.740166000	-0.000000000	-0.000000000
B	-2.307326000	-1.332135000	0.000000000
C	1.370083000	-2.373053000	-0.000000000
C	-2.740166000	0.000000000	-0.000000000
B	2.307326000	-1.332135000	0.000000000
B	-2.307326000	1.332135000	0.000000000

C	1.370083000	2.373053000	-0.000000000
B	-0.000000000	-2.664270000	0.000000000
C	-1.370083000	2.373053000	-0.000000000
Ca	0.000000000	0.000000000	0.000000000
C	-1.370083000	-2.373053000	-0.000000000
B	2.307326000	1.332135000	0.000000000

$Ca©B_6C_6$
HF = -1055.1837436 a.u.
Frequencies v_1= 47 cm^{-1}

B	0.000000000	2.581590000	0.698290000
C	0.000000000	2.594799000	-0.714998000
B	-0.000000000	-0.797413000	2.699651000
B	0.000000000	0.797413000	2.699651000
B	-0.000000000	-2.581590000	0.698290000
C	0.000000000	-0.617283000	-2.567298000
C	-0.000000000	-1.926082000	1.942830000
Ca	-0.000000000	0.000000000	0.045811000
C	0.000000000	1.926082000	1.942830000
B	0.000000000	1.914237000	-1.882205000
C	0.000000000	0.617283000	-2.567298000
C	-0.000000000	-2.594799000	-0.714998000
B	-0.000000000	-1.914237000	-1.882205000

$Sr©B_8C_5^-$
HF = -419.8992256 a.u.
Frequencies v_1= 62 cm^{-1}

Sr	0.000000000	0.000000000	0.040352000
B	-0.000000000	2.715751000	-1.036270000
B	0.000000000	-2.494638000	1.606270000
B	-0.000000000	1.375493000	2.729170000
B	0.000000000	-0.801154000	-2.901740000
B	-0.000000000	-2.715751000	-1.036270000
B	0.000000000	2.494638000	1.606270000
B	0.000000000	0.801154000	-2.901740000
B	-0.000000000	-1.375493000	2.729170000
C	0.000000000	-2.997513000	0.338342000
C	-0.000000000	1.983011000	-2.248665000
C	-0.000000000	-1.983011000	-2.248665000
C	0.000000000	0.000000000	2.902699000
C	0.000000000	2.997513000	0.338342000

$Sr©BC_{13}^+$
HF = -532.1771979 a.u.
Frequencies v_1= 33 cm^{-1}

C	0.000000000	2.759364000	-0.672074000
C	0.000000000	2.823940000	0.599443000
C	0.000000000	2.255686000	-1.858063000
C	0.000000000	2.251306000	1.763851000
C	0.000000000	1.230727000	-2.621337000

C	0.000000000	1.342339000	2.649699000
C	0.000000000	0.000000000	-2.982522000
B	0.000000000	0.000000000	3.043062000
Sr	0.000000000	0.000000000	0.114252000
C	-0.000000000	-2.759364000	-0.672074000
C	-0.000000000	-2.823940000	0.599443000
C	-0.000000000	-2.255686000	-1.858063000
C	-0.000000000	-2.251306000	1.763851000
C	-0.000000000	-1.230727000	-2.621337000
C	-0.000000000	-1.342339000	2.649699000

Ba©$B_4C_{10}^-$
HF = -505.7473473 a.u
Frequencies v_1= 14 cm^{-1}

B	0.000000000	1.320202000	2.726672000
C	0.000000000	0.000000000	-2.956284000
B	0.000000000	-2.319785000	-1.870577000
C	0.000000000	1.285009000	-2.851736000
B	0.000000000	2.319785000	-1.870577000
C	0.000000000	-2.473621000	1.866930000
C	-0.000000000	-1.285009000	-2.851736000
B	-0.000000000	-1.320202000	2.726672000
C	0.000000000	2.473621000	1.866930000
C	0.000000000	-2.969425000	-0.654886000
C	0.000000000	0.000000000	3.140815000
C	0.000000000	-2.844056000	0.655579000
Ba	0.000000000	0.000000000	0.038236000
C	0.000000000	2.969425000	-0.654886000
C	0.000000000	2.844056000	0.655579000

Ba©$B_3C_{12}^+$
HF = -569.919621077
Frequencies v_1= 26 cm^{-1}

C	-3.090063000	-0.359824000	0.000000000
C	-2.800581000	-1.648806000	0.000000000
C	-3.139791000	0.889266000	0.000000000
C	-1.817296000	-2.449738000	0.000000000
B	-2.403280000	2.110309000	0.000000000
C	-0.646637000	-3.001762000	0.000000000
C	-1.264582000	2.888015000	0.000000000
Ba	-0.000000000	0.098379000	0.000000000
C	3.146418000	0.868068000	-0.000000000
C	3.088039000	-0.380902000	-0.000000000
B	2.417139000	2.093249000	-0.000000000
C	2.789813000	-1.667349000	-0.000000000
C	1.283570000	2.878395000	-0.000000000
C	1.801995000	-2.463014000	-0.000000000
C	0.009979000	3.032936000	0.000000000
C	0.627589000	-3.006458000	-0.000000000

$Ba©B_2C_{13}^+$
HF = -556.726330178
Frequencies v_1= 33 cm^{-1}

C	0.000000000	1.844777000	-2.626891000
C	0.000000000	2.732828000	-1.774360000
C	0.000000000	0.614129000	-3.158497000
B	0.000000000	3.212483000	-0.408527000
C	-0.000000000	-0.614129000	-3.158497000
C	-0.000000000	3.000230000	0.929558000
C	-0.000000000	2.289652000	2.020715000
Ba	-0.000000000	0.000000000	0.157070000
C	-0.000000000	-3.000230000	0.929558000
C	-0.000000000	-2.289652000	2.020715000
C	-0.000000000	-1.360716000	2.879388000
B	-0.000000000	-3.212483000	-0.408527000
B	-0.000000000	0.000000000	3.210081000
C	-0.000000000	-2.732828000	-1.774360000
C	0.000000000	1.360716000	2.879388000
C	-0.000000000	-1.844777000	-2.626891000
